# Proteomic Identification of an Upregulated Isoform of Annexin A3 in the Spinal Cords of Rats in a Neuropathic Pain Model

**DOI:** 10.3389/fnins.2017.00484

**Published:** 2017-09-05

**Authors:** Wangyuan Zou, Wei Xu, Zongbin Song, Tao Zhong, Yingqi Weng, Changsheng Huang, Maoyu Li, Chuanlei Zhang, Xianquan Zhan, Qulian Guo

**Affiliations:** ^1^Department of Anesthesiology, Xiangya Hospital, Central South University Changsha, China; ^2^Key Laboratory of Cancer Proteomics of Chinese Ministry of Health, Xiangya Hospital, Central South University Changsha, China; ^3^Hunan Engineering Laboratory for Structural Biology and Drug Design, Xiangya Hospital, Central South University Changsha, China

**Keywords:** neuropathic pain, spinal cord, proteomics, ANXA3, microglia

## Abstract

Neuropathic pain (NP) is induced by nerve damage or a disturbance in the peripheral or central nervous systems. Nerve damage causes the activation of sensitizing mechanisms in the peripheral and central nervous systems, which induces transcriptional and post-transcriptional alterations in sensory nerves. However, the underlying mechanisms of NP remain elusive. In the study, Two-dimensional gel electrophoresis (2DGE)-based comparative proteomics identified 38 differential gel spots, and 15 differentially expressed proteins (DEPs) between the sham and the chronic constriction injury (CCI)-induced neuropathic pain rats. Of them, Annexin A3 (ANXA3) was significantly increased after CCI with Western blot analysis and immunofluorescence imaging. A lentivirus delivering ANXA3 shRNA (LV-shANXA3) was administered intrathecally to determine the analgesic effects of ANXA3 on allodynia and hyperalgesia in a CCI-induced neuropathic pain model in rats. Further study showed that LV-shANXA3 reversed the upregulation of ANXA3, alleviated CCI-induced mechanical allodynia and thermal hyperalgesia. The study indicated that ANXA3 may play an important role in neuropathic pain.

## Introduction

Neuropathic pain (NP) is induced by nerve damage or a disturbance in the peripheral or central nervous systems, and is often accompanied with allodynia and hyperalgesia (Zimmermann, 2001). However, the molecular mechanisms of neuropathic pain remain unclear, and the treatments for patients of neuropathic pain are not ideal. Therefore, it is necessary and urgent to determine new therapeutic targets for neuropathic pain (Niederberger and Geisslinger, 2008; Kuhlein et al., 2011; Rouwette et al., 2016).

Proteomic analysis in animals with neuropathic pain can be helpful in the determination of pain-related proteins that could be potential therapeutic targets for patients suffering from neuropathic pain. Some proteomic studies (Alzate et al., 2004; Sung et al., 2004; Kunz et al., 2005; Komori et al., 2007; Sun et al., 2007; Fujisawa et al., 2008; Singh et al., 2009; Rouwette et al., 2016) have been performed in a variety of animal models such as spinal nerve ligation (SNL), chronic constriction injury (CCI) of the sciatic nerve, and the spared nerve injury (SNI) neuropathic pain models. The high throughput screening studies reported many DEPs may be associated with the development and maintenance of NP (Sui et al., 2014; Vacca et al., 2014; Casas et al., 2015). Our previous study revealed that some differentially expressed proteins (DEPs) may be involved in the pro-nociceptive role of PKCγ in CCI-induced neuropathic pain (Zou et al., 2012).

2DGE-based comparative proteomics was employed to determine new protein targets in CCI-induced NP. The protein in every gel-spot was distinguished by MALDI-TOF peptide mass fingerprint (PMF) examination, by which we identified dozens of DEPs, including Annexin A3 (ANXA3). ANXA3 is a protein that is encoded by humans ANXA3 gene, which, in turn, encodes a part of the Annexin family. Many members in this calcium-dependent phospholipid-binding protein family perform a function in the control of cellular development and signal transduction pathways, but their physiological function are mainly unclear. It was reported that ANXA3 might be a Ca^2+^-dependent advocate amid phospholipids and F-actin in microglia up-regulated induced by peripheral nerve damage (Konishi et al., 2006). Evidence from other studies indicates that ANXA3 plays an important role in cancer, and might be a bio-marker and possible drug target for neoplastic breast cell, blockade of ANXA3/JNK signaling pathway provides a therapeutic target for hepatocellular carcinoma (Wu et al., 2013; Pan et al., 2015; Tong et al., 2015; Zeidan et al., 2015), and some reports indicate that ANXA3 is a biomarker of some cancers (Kollermann et al., 2008; Leman and Getzenberg, 2009; Liu et al., 2009). However, no study has revealed the role of ANXA3 in CCI-induced neuropathic pain, so we hypothesized that ANXA3 plays potential vital role in neuropathic pain in rats.

## Materials and methods

### Animal preparations

Male Sprague-Dawley rats (weighing between 200 and 250 g, age between 7 and 8 weeks old) were bought from the Experimental Animal Center of Central South University. All of the rats were kept separately in cages (20 ± 0.5°C; 12-h light/dark cycle) and *ad libitum* access to food and water. All of the animals' experimental methods and procedure were authorized by the Central South University Animal Care and Use Committee (Zou et al., 2012), and followed the Ethical Guidelines of the International Association for the Study of Pain (Zimmermann, 1983).

### Chronic constriction injury (CCI)-induced neuropathic pain model

The process that produces the CCI neuropathic pain model was reproduced as previously described (Bennett and Xie, 1988). In brief, animals were anesthetized with phenobarbital sodium (35–50 mg/kg, i.p.). The left sciatic nerve was revealed by blunt dissection. Situated near the sciatic trifurcation, 4 ligatures (4-0 chromic gut) with ~1-mm of space were not tightly surrounded the sciatic nerve. A sham operation was executed with the sciatic nerve revealed but not ligated. The animals that had undergone the CCI procedure and demonstrated obvious mechanical and thermal hypersensitivity were used for further behavioral experiments.

### Measurement of pain threshold

Sprague-Dawley rats were randomly divided into four groups as follows: Sham, CCI+Normal Saline (NS), CCI+LV-NC (LV-NC), CCI+LV-shANXA3 (LV-shANXA3), *n* = 8 animals in each group. Behavioral assessment was accomplished using the same experimenters blinded to the treatment. The animal was put in a plexiglass compartment on a raised mesh partition. Behavioral acclimation was permitted for 30 min. Paw mechanical withdrawal thresholds (PMWT) were ascertained in reaction to pressure as described (Chaplan et al., 1994). The amount of pressure (g) required to generate a paw withdrawal response was calculated three times on each paw after 3 min intermissions. The results of 3 tests per each paw per day were averaged. PMWT was calculated (1 day prior to CCI as a baseline; and on the third, fifth, seventh, tenth, and fourteenth days after CCI).

Paw withdrawal thermal latency (PWTL) was detected with a Hargreaves instrument (Plantar test, 7370, Ugo Basile, Italy) as previously described (Hargreaves et al., 1988). The animals were put in clear plastic cages on a raised glass plate and permitted to become accustomed to the new environment for 30 min prior to evaluation. After acclimation, a luminous heat origin of constant intensity was placed underneath the glass and aimed at the mid-plantar area. A digital timer mechanically read the span between the initiations of stimuli and paw removal. The PWTL was quantified to the closest 0.1 s. The baseline PWTL of the animals was managed at ~11 s by adapting the strength of the light on the pain threshold detector. A cutoff period of 15 s of irradiation was implemented to prevent any tissue injury. Five minutes was allowed to elapse between stimulations. In the subsequent behavioral experiments, PMWT and PWTL were calculated 1 day prior to CCI (baseline) and 3, 5, 7, and 14 days (8, 10, 12, and19 days after CCI) after intrathecal lentivirus or normal saline administration.

### Western blot analysis

Dissected L4-5 spinal cords of sham, CCI, LV-NC, and LV-shANXA3 treated rats were cleaned using cold phosphate-buffered saline with 2 mM of EDTA and lysed with a denaturing SDS-PAGE sample buffer via conventional techniques. Protein lysates were divided with a 10% SDS-PAGE and the proteins were shifted onto a PVDF membrane (Millipore, Bedford, MA, USA). The PVDF membranes with proteins were blocked using TBS with 0.1% Triton X-100 and 5% skim milk (overnight; 4°C), and were incubated (overnight; 4°C) with diluted rabbit anti-ANXA3 antibody (1:300, Cell Signal Technology, USA). After TBS cleaning three times, the membranes were incubated (room temperature; 2 h) with diluted (1:5000) HRP-conjugated goat anti-rabbit IgG antibody (Abcam, USA). The protein was inspected using an ECL system (Amersham Biosciences, USA). β-tubulin antibody (Multisciences, China, and 1:2,000 dilution) was probed as a loading control. The Western blots were digitalized using a camera. The ratio of ANXA3 to tubulin was determined by densitometry evaluation. All Western blot analysis (the same biological sample) was repeated at least three times.

### Two-dimensional gel electrophoresis (2DGE), gel scanning, and image analysis

The L4-5 spinal cord samples of animal tissues (Sham and CCI-treated rats) were prepared at day 7 after CCI and 2DGE and gel scanning were performed as previously described (Zou et al., 2012). The removed proteins were counted with a 2-D Quantification kit (Amersham Biosciences, Sweden). The proteins of spinal cords were separated with 2DGE as previously described (Zou et al., 2012). Briefly, each protein sample (600 μg) was diluted and put on IPG strips (pH 3–10 NL, 24 cm) for 14 h of rehydration at 30 V. The proteins were divided based on the pI under the state (1 h at 500 V, 1 h at 1,000 V, and 8.5 h at 8,000 V to assemble 68 kVh) on an IPGphor (Amersham Biosciences). Aimed proteins in IPG strips were equilibrated for 15 min in a mixture (6 M of urea, 2% SDS, 30% glycerol, 50 mM of Tris-HCl, pH 8.8, and 1% DTT), and alkalized for another 15 min in a mixture (6 M of urea, 2% SDS, 30% glycerol, 50 mM of Tris-HCl, pH 8.8, and 2.5% iodoacetamide). The aimed proteins in the IPG strips were separated based on the M_*r*_ with SDS-PAGE (12%) on the Ettan DALTII system (Amersham Biosciences). The Coomassie blue G-250 staining was implemented to view the 2DGE-separated proteins. We pull all the lumber spinal cord protein samples of each group together. 2DGE of each group was done in triplicate.

### Gel scanning and image analysis

The visualized 2-D gels were scrutinized by MagicScan software on an Imagescanner (Amersham Biosciences). The 2-D gel images were evaluated by a PDQuest software (Bio-Rad Laboratories, Hercules, CA). Duplicate gels were prepared for each tissue. The gel spot design of every gel was recapped in traditional after-spot matching. A standard gel was gathered for every tissue. Spot density were measured by spot-volume [O.D. × (I.U.)^2^]. Every spot-volume in a gel was normalized using the total spot-volume of the gel. The normalized spot-volume was implemented to identify every DEP with a difference ≥two-fold in the CCI group in contrast to the sham group. Statistical significance was determined by student's *t*-test that had a level of 0.05.

### In-gel trypsin digestion

The gel spots that had differentially conveyed proteins were removed from the 2-D gels. The proteins that were contained in every gel-spot were forced into in-gel trypsin digestion (Sun et al., 2007; Zou et al., 2012) and handled for mass spectrometric analysis. In short, the removed gel spots were dehydrated via sonication for 20 min in 100 μl of mixture with 50% (v/v) actonitrile and 100 mM of ammonium bicarbonate until the gel turned opaque white, and were shrunken in 50 μl of actonitrile for 10 min. After the extraction of residual solvent, the dehydrated gels were rehydrated in 50 mM of NH_4_HCO_3_, pH 7.8, holding 0.5 μg of modified trypsin (Promega, Madison, WI, USA). The rehydrated gels were kept overnight at 37°C for protein digestion with trypsin. The tryptic peptides were extracted using 0.1% formic acid in 50% acetonitrile and desalted and condensed with ZipTips that had C18 resin (Millipore, Bedford, MA).

### MS-identification of protein

The MS-identification of protein is the same as previously reported (Zou et al., 2012). The mass spectrometric (MS) spectra were gained on a Voyager DE STR MALDI-TOF mass spectrometer (ABI, Foster City, CA). The MS spectra were handled with baseline correction, noise elimination (5%), and peak deisotoping with the Mascot Wizard (Matrix Science; http://www.matrixscience.com/) to gather PMF information. The PMF information was input into Mascot for protein identity scrutinizing.

The PMF information from the Voyager DE STR mass spectrometer was scrutinized against the Swiss-Prot protein database (version 57.1), which has 462,764 sequences. The query taxonomy was limited to “Rattus” Proteins that had score larger than 51 were considered as significance (*p* < 0.05), and only the Rattus proteins with the highest results in every Mascot query were taken as fruitful recognitions. The treatment of amino acid sequence, the Mascot results, the relative mass, and pI that were produced from gels, the computed mass and pI that were generated from the database, and Swiss-Prot accession number are obtained for every protein.

### Immunohistochemical staining

The animals were deeply anesthetized with phenobarbital sodium (60 mg/kg, i.p.). and perfused transcardially with 100 ml of PBS and then by 250 ml of ice-cold 4% paraformaldehyde. The lumbar (L4–5) segment of the spinal cord was carefully removed, and fixed with 4% paraformaldehyde/PBS (Sigma, US) and dehydrated in 70% ethanol. The fixed tissues were infiltrated in 30% sucrose in PBS at 4°C overnight. Transverse sections (20 mm) of the L4–5 lumbar spinal cord were incubated for 2 h at room temperature in an obstructing mixture (3% normal goat serum) and immunohistochemical analysis was performed using anti-ANXA3 (dilution of 1:300, Cell Signal Technology, US), anti-NeuN (1:300, mouse, Millipore, US), anti-GFAP (1:800A,bcam, mouse, US), and anti-IBa1 (1:400, goat, Abcam, US) antibodies. The controls were routinely established by incubation with rabbit IgG. The GFP fluorescence in the spinal cord cells was recorded with a fluorescent microscope (Leica, Germany).

### Intrathecal catheter implantation

Lumbosacral intrathecal catheters were inserted as described by Yaksh (Malkmus and Yaksh, 2004). LV-shANXA3, LV-NC control vector, or normal saline (NS) were injected intrathecally on day 5 after CCI. All intrathecal microinjections were performed using a 10 μl volume to guarantee total drug delivery. Every one of the catheter arrangements were confirmed after death by optical examination. Only data from animals that had tips that had entered the intrathecal catheter at the lumbar enlargement of the spinal cord were analyzed.

### Lentiviral vectors

Lentiviral vectors pGCSIL-shRNA-GFP (LV-shANXA3 and LV-NC) were produced as previously described (Coleman et al., 2003; Zou et al., 2011; He et al., 2013). The recombinant lentivirus containing ANXA3 shRNA (LV-shANXA3) or NC shRNA (LV-NC) was packaged using a pGCSIL-GFP vector by Shanghai GeneChem. Sense siRNA sequences targeting ANXA3 (GenBank accession NM_012823) and control sequences were as follows: shANXA3, CTCTACGATGCTGGTGAGAAA; Scrambled shRNA (NC) siRNA, TTCTCCGAACGTGTCACGT. The last titer of the recombinant virus was 5 × 10^8^ TU/ml by ultracentrifugation. LV vector-only expressing GFP was used as the negative control (LV-NC).

### Statistical analysis

Data are presented as mean ± standard deviation (*SD*) of the indicated number of separate experiments. All statistical comparisons were computed using SPSS 19.0. Time-course measures for each behavioral test were analyzed by repeated-measures of ANOVAs followed by Bonferroni test. The statistical software package SPSS 19.0 was used in this study. For 2D gel image analysis and Western blot analysis, the relative density of each protein was calculated by dividing the optical density value of each protein by the optical density of the corresponding loading control, and Student's *t*-test and one-way analysis of variance (ANOVA) were used to determine the Differences were deemed significant at *P* < 0.05.

## Results

### CCI-induced mechanical hypersensitivity

**Figure 1:**
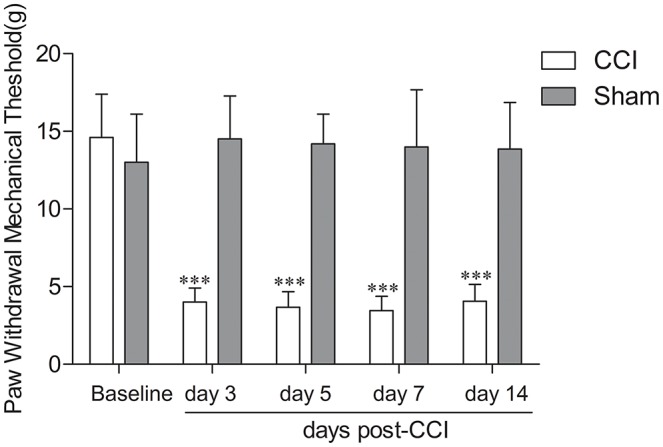
Chronic constriction injury (CCI)-induced mechanical allodynia in rats. CCI resulted in a significant decrease in paw withdrawal threshold in response to mechanical stimulation of the injury on the paw side on day 3, 5, 7, and 14 post surgery in rats (*n* = 8); ^***^P < 0.001, compared with the baseline and sham group (*n* = 8). Sham surgery did not cause mechanical hypersensitivity (*n* = 8; *P* > 0.05).

No differences were observed between the groups with respect to weight. To evaluate mechanical allodynia in CCI rats, PWMT evoked by mechanical stimuli was determined using von Frey filaments. As shown in Figure 1, the results demonstrated a substantial decline in PWMT on ipsilateral hind paw on Days 3, 5, 7, and 14 after CCI surgery compared with the baseline and the sham group (Figure 1, *n* = 8; *P* < 0.001). Rats who received sham surgeries did not show mechanical hypersensitivity (Figure 1, *n* = 8; *P* > 0.05).

### Determination of DEPs in the spinal cord of CCI-induced neuropathic pain rats

**Figure 2:**
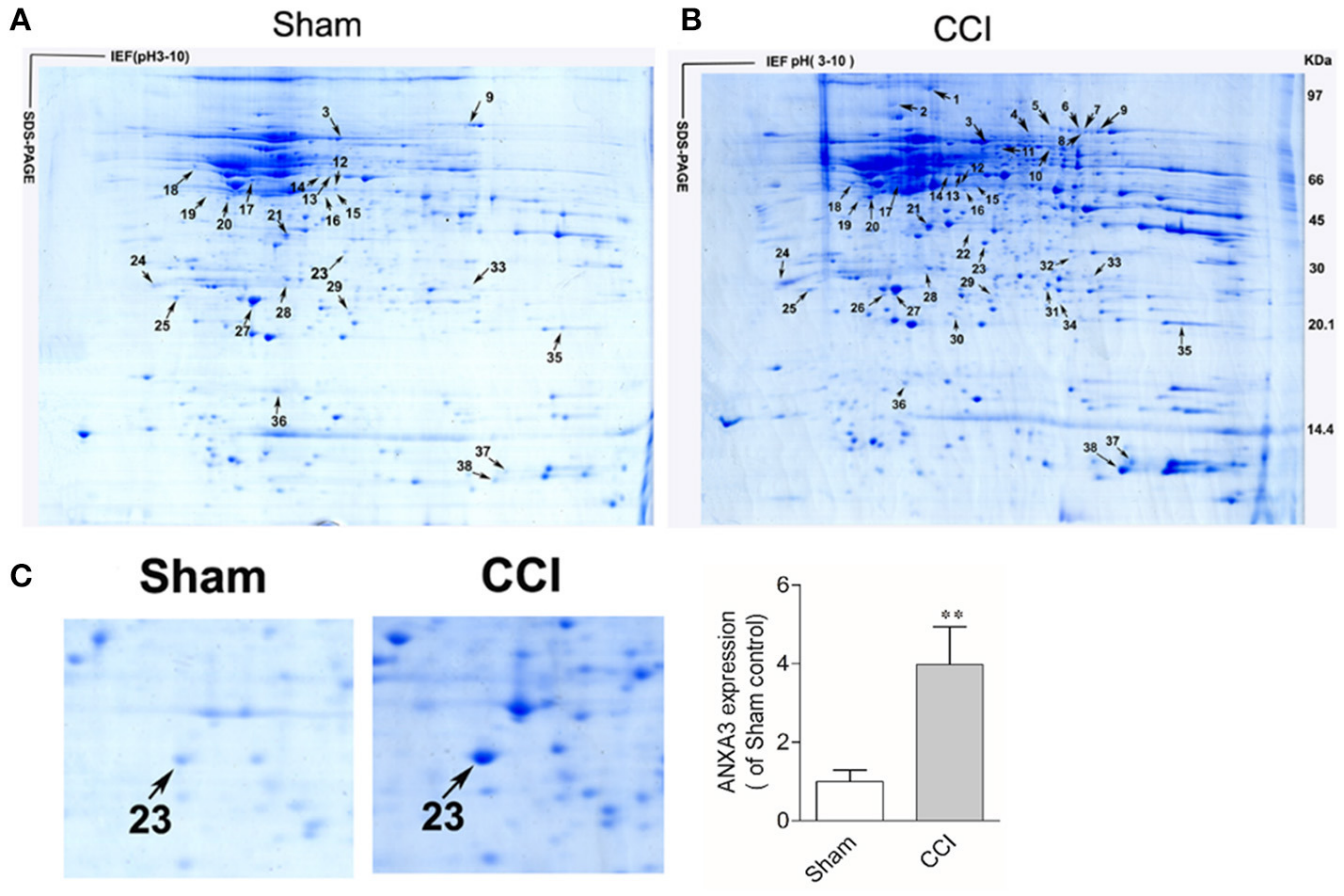
Coomassie brilliant blue G250 stained 2-D gels of spinal cord proteins of the sham **(A)**, and CCI **(B)** groups, respectively. Sixteen DEP-spots are marked with arrows. The first dimension, an isoelectric focusing (IEF), was performed with immobilized pH gel (pH 3–10), as indicated on the horizontal axis. The second dimension, SDS-PAGE, was performed with a 9~18% SDS polyacrylamide gel, where the proteins are separated by their molecular weight, as indicated on the vertical axis. Arrows refers to MS-identified DEPs. The outcomes are displayed in Table 1. **(C)** Close-up image of partial DEP-spots between the sham and CCI groups. (^**^P < 0.01, *n* = 4 animals per group).

**Figure 3:**
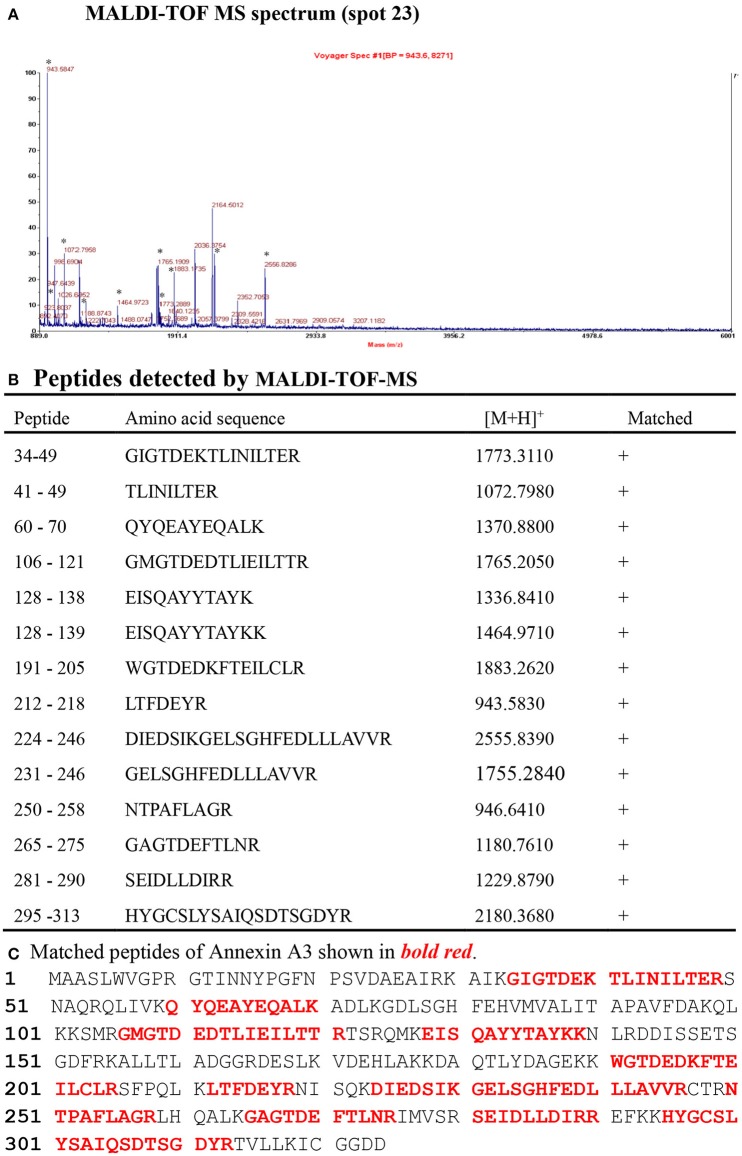
Identification of ANXA3 (Spot 23) with MALDI-TOF-MS peptide mass fingerprint.

Two representative 2DGE maps from the sham and CCI groups were shown in Figures 2A,B. To ensure a reliable evaluation of protein expression, 2DGE from every group was executed for three times; the 2DGE designs were well-resolved and optically duplicated, with 38 recognized differential gel-spots. DEPs were removed for MALDI-TOF-MS PMF evaluation. Fifteen DEPs were determined and observed in all samples by high-quality MS spectra (Supplementary Presentation 1). A focused image of the gel areas revealing DEPs, a significantly upregulated protein spot (Spot-23), between the sham and CCI group is displayed in Figure 2C. The MALDI-TOF MS spectrum, the determined peptides, and the marched protein in representative spot 23 (ANXA3) are shown in Figure 3.

Identified proteins were significantly upregulated or downregulated (≥two-fold; *P* < 0.05) in the CCI. All of these proteins are listed in Table 1 and are associated with the correlating 2-D gel-spot in Figures 2A,B. Swiss-Prot protein notation sheet showed the DEPs were involved in various biological roles, such as heat shock proteins, chaperones, antioxidant proteins, proteins involved in signal transduction, cytoskeleton protein, biomarker of glia and neuron, proteins required for cellular homeostasis and metabolism, and proteins needed for plasma membrane receptor trafficking.

**Table 1:** Differentially expressed proteins in the spinal cords between CCI-NP and Sham rats, identified with 2DGE-based comparative proteomics.

**Spot no**	**Accession no**	**Protein name**	**MW (Da)**	**pI**	**Score**	**Coverage (%)**	**Expression**
**HEAT SHOCK PROTEINS, CHAPERONES, AND ANTIOXIDANT PROTEINS**							
2	HSP74_RAT	Heat shock 70 kDa protein 4	94,795	5.13	88	13	+
27	PRDX1_RAT	Peroxiredoxin-1	22,323	8.27	53	24	↑
35	PRDX1_RAT	Peroxiredoxin-1	22,323	8.27	97	44	↑
**PROTEINS INVOLVED IN SIGNAL TRANSDUCTION**							
**23**	**ANXA3_RAT**	**Annexin A3**	**36,569**	**5.96**	**55**	**23**	↑↑
**CYTOSKELETON PROTEIN**							
1	SPTA2_RAT	Spectrin alpha chain	285,261	5.20	63	6	+
38	HBA_RAT	Hemoglobin subunit alpha-1/2	15,490	7.82	58	32	↑↑
**BIOMARKER OF GLIA AND NEURON**							
17	GFAP_RAT	Glial fibrillary acidic protein	49,984	5.35	86	23	↑↑
14	NFM_RAT	Neurofilament medium polypeptide	95,848	4.77	84	18	↑
18	NFL_RAT	Neurofilament light polypeptide	61,355	4.63	82	22	↑
**PROTEINS INVOLVED IN CELLULAR HOMEOSTASIS AND METABOLISM**							
3	ALBU_RAT	Serum albumin	70,682	5.06	157	34	↑↑
7	ACON_RAT	Aconitate hydratase	86,121	7.87	139	26	↑
21	GBB2_RAT	Guanine nucleotide-binding protein G(I)/G(S)/G(T) subunit beta-2	38,048	5.60	116	68	↑
22	MDHC_RAT	Malate dehydrogenase	36,631	6.16	72	26	+
28	6PGL_RAT	6-phosphogluconolactonase	27,445	5.54	71	31	↓
**PROTEINS INVOLVED IN PLASMA MEMBRANE RECEPTOR TRAFFICKING**							
25	SNAP25_RAT	Synaptosomal-associated protein, 25 KDa	23,528	4.66	54	41	↑

*Up-arrows = upregulated. Down-arrows = downregulated. 2DGE of each group was done in triplicate. (n = 4 animals per group). Plus sign = New DPEs in the CCI-NP rats. Bold values mean to highlight the classification of DEPs and to highlight Annexin A3*.

### ANXA3 is expressed in the microglia of the spinal cord

**Figure 4:**
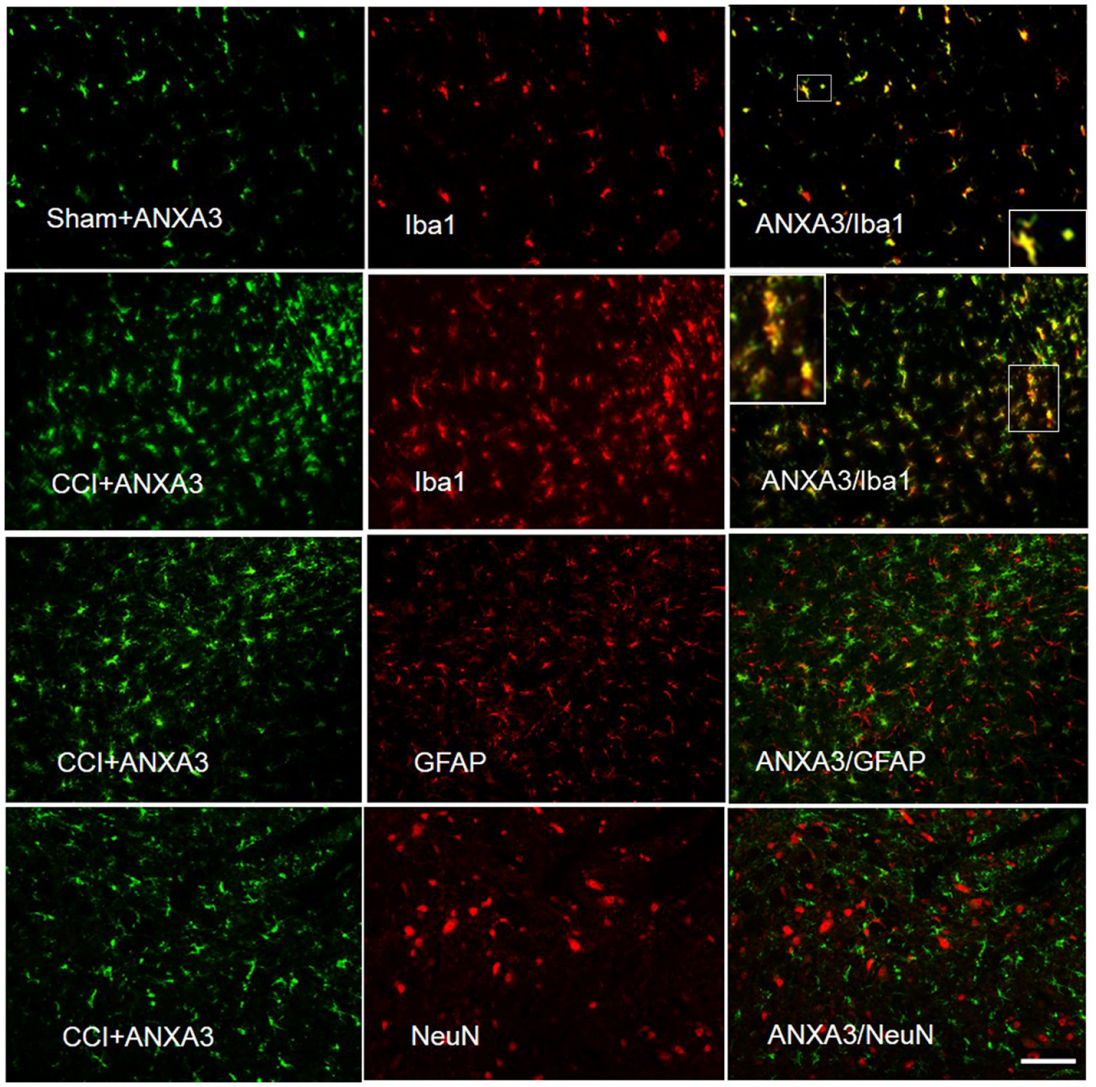
Cell specific localization of Annexin A3 in the spinal cord of sham treated or CCI rats as assessed by immunofluorescence staining of the spinal cord for Annexin A3 (green), neurons using NeuN antibody (red), astrocytes using GFAP antibody (red), and microglia using Iba1 antibody (red). ANXA3 was co-expressed with the microglia in the lumber spinal cord. Scale bar = 50 μm.

To identify ANXA3-positive cells and obtain further information on its cellular role, we executed double immunofluorescent staining with Iba1, the microglia specific marker, GFAP, an astrocyte marker, and NeuN, the neuronal nuclei-specific antibody, in the L4-5 spinal cord of CCI rats. Immunostaining showed that ANXA3 was expressed in the microglia of the lumber spinal cord (Figure 4).

**Figure 5:**
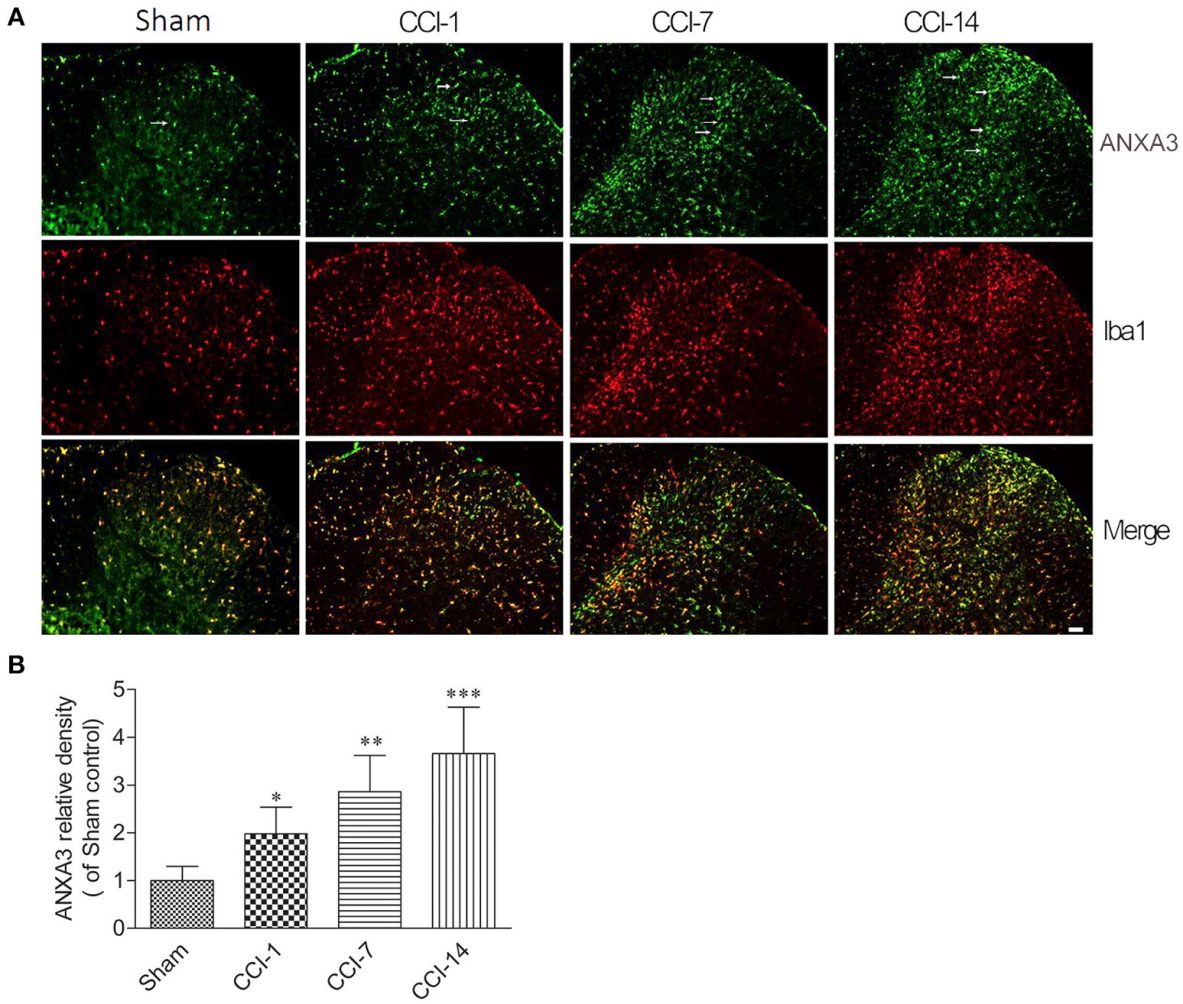
**(A)** The dynamic expression changes of ANXA3 in the dorsal horn of the spinal cord as assessed by immunofluorescence staining for Annexin A3 (green) and microglia using Iba1 antibody (red). **(B)** Compare with the sham group, the expression of ANXA3 in the dorsal horn of the spinal cord was increasing from day 1 to day 14 after CCI. Scale bar = 50 μm (^*^P < 0.05, ^**^P < 0.01, ^***^P < 0.001, *n* = 5 animals per group).

We further explored the dynamic expression changes of ANXA3 in the dorsal horn of the spinal cord. As shown in the Figures 5A,B, compare to the sham group, the expression of ANXA3 in the dorsal horn of the spinal cord was increasing from day 1 to day 14 after CCI (*n* = 5; *P* < 0.05).

### Intrathecal injection of LV-shANXA3 reduced ANXA3 expression in the spinal cord

**Figure 6:**
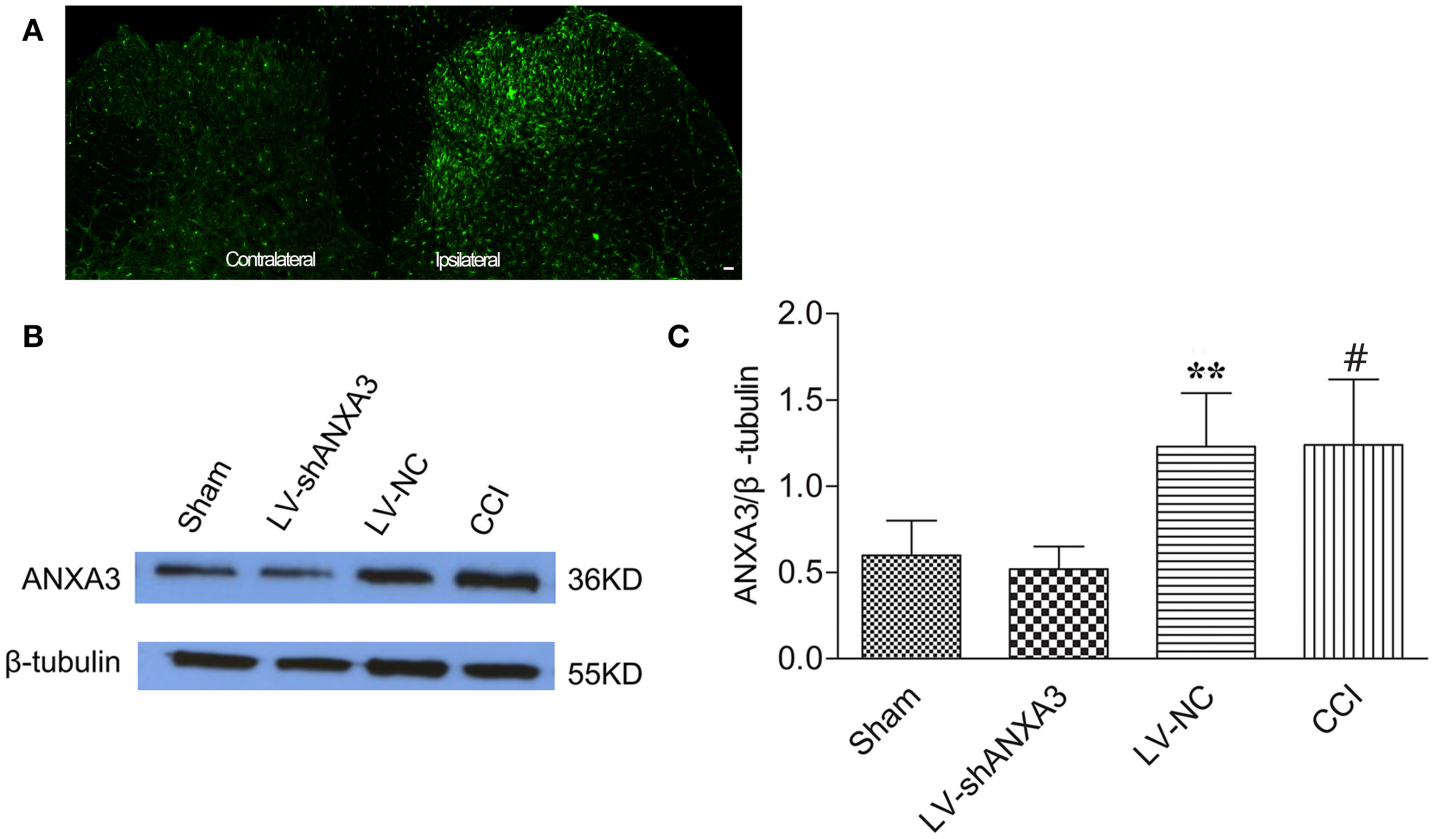
ANXA3 expression in the spinal cord after intrathecal delivery of LV-NC and LV-shANXA3. **(A)** Staining showed that the expression of ANXA3 in the dorsal horn of spinal cord in the ipsilateral was upregulated compared with the contralateral side. **(B,C)** Western blot analysis demonstrated that the ANXA3 protein was downregulated by LV-shANXA3 (10 μl) 7 days after intrathecal delivery in CCI-induced neuropathic pain rats compared with the sham and the LV-NC control rats. β-tubulin was used as the loading control. Each experiment was performed triplicate with indicated number of sample. Significance is defined as ^**^P < 0.01 compared with LV-NC negative vector control group, ^#^*P* < 0.01 compared with the sham control group (*n* = 5 animals per group).

Animals were scrutinized after intrathecal injection of lentiviral vectors and showed normal motion and diet. As shown in Figure 6A, the expression of ANXA3 in the dorsal horn of spinal cord in the ipsilateral of rats was upregulated compared with the contralateral side. As shown in Figures 6B,C, the expression of ANXA3 in the spinal cord was substantially upregulated in CCI group when compared with the sham rats, whereas LV-shANXA3 treatment significantly reversed the upregulation of ANXA3 expressions and LV-NC treatment did not show such effect (*P* = 0.0005, Figure 6). β-tubulin was utilized as the loading control. Compared to the sham and the LV-NC control groups, Western blot analysis demonstrated that ANXA3 protein was downregulated by LV-shANXA3 (10 μl) treatment 7 days post-intrathecal injection in CCI rats.

### Lentivirus-mediated ANXA3 shRNA attenuated tactile allodynia and thermal hyperalgesia

**Figure 7:**
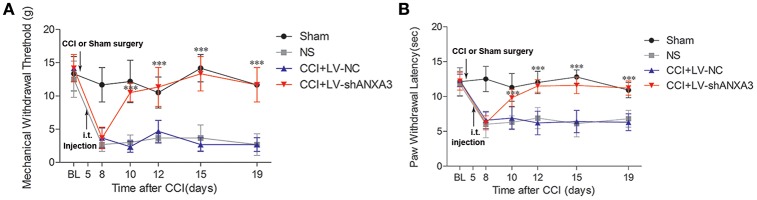
Tactile allodynia and thermal hyperalgesia after CCI and intrathecal injection of LV vectors. Animals were either left un-operated (sham) or injected with saline (NS), ANXA3 shRNA negative control vectors (LV-NC), or LV-shANXA3 vectors (LV-shANXA3) for a total volume of 10 μl, respectively. Only animals that demonstrated tactile allodynia and thermal hyperalgesia 5 days after CCI were injected with the vectors. **(A)** PMWT and **(B)** PWTL were measured 1 day before CCI (BL, baseline) and 8, 10, 12, 15, and 19 days after CCI or 3, 5, 7, 10, and 14 days after intrathecal delivery. “BL” is the time point of the basic pain threshold before CCI in rats, “8” is the time point 8 days after CCI (3 days after intrathecal delivery). Data are expressed as mean ± *SD, n* = 8, Significance is defined as ^*^P < 0.05, ^***^P < 0.001 compared with LV-NC at each time point by one-way ANOVA followed by Bonferroni test.

Following injection of the lentiviral vectors on day 5 after CCI-treated rats, the animals had a normal appearance, and normal ability of activity, and ate regularly. To further investigate whether ANXA3 plays a role in neuropathic pain, lentiviral vectors mediated shANXA3 (LV-shANXA3, 10 μl) or controlled negative vectors (LV-NC, 10 μl) were injected intrathecally in CCI-induced neuropathic pain rats. Compared with LV-NC or NS treatment rats, LV-shANXA3 alleviated mechanical allodynia from day 5 to 14 (day 10 to 19 after CCI; *P* < 0.001, Figure 7A, *n* = 8) and heat hyperalgesia (*P* < 0.001, Figure 7B, *n* = 8) from day 5 to 14 after injection, which corresponded with the decreased ANXA3 protein expression revealed by Western blot analysis (Figures 6B,C).

## Discussion

Nerve injury-induced neuropathic pain is a large public health issue all over the world with limited treatment options. This study is the initial research showing that ANXA3, from some determined proteins, was greatly upregulated in the spinal cord following CCI-induced neuropathic pain. In particular, ANXA3 is an upregulated protein that is located in the microglia of the spinal cord dorsal horn and contributes to neuropathic pain.

Previously proteomic evaluations from spinal cords or DRG tissues discovered changes in the expression of different proteins in CCI-induced neuropathic pain models (Niederberger and Geisslinger, 2008; Kamagata et al., 2011; Gao et al., 2012, 2013). In this study, we determined changed proteins including in heat shock proteins, chaperones, and antioxidant proteins, signal transduction, biomarkers of glia and neurons, cellular homeostasis and metabolism, cytoskeleton protein, and plasma membrane receptor trafficking. Some of the proteins were reported in previously proteomic evaluations using multiple nerve injury models (Kunz et al., 2005; Komori et al., 2007; Niederberger and Geisslinger, 2008; Singh and Tao, 2012; Rouwette et al., 2016), which indicated that different proteins may contribute to different nerve injury neuropathic pain models. The findings on expression of ANXA3 and GFAP were consistent with other proteomics studies (Komori et al., 2007), but most of the protein spots were inconsistent, which may due to the variance in collected tissues, time points and animal models.

In this study, proteomics evaluation showed ANXA3 as a candidate protein present in nerve damage. It was the first time that researchers discovered an upregulated expression in ANXA3 expression in the spinal cord following CCI-induced neuropathic pain in rats. We found abundant expression of ANXA3 in the microglia of the spinal cord. Intrathecal injection of LV-shANXA3 alleviated the CCI-induced hyperalgesia and allodynia, which indicates that ANXA3 are crucial in neuropathic pain. Some studies reported that ANXA3 was a novel marker of brain microglia (Junker et al., 2007) and ANXA3 is produced by activated microglial cells. And Iba1-/ANXA3-immunopositive cells may play an important role in clearing the inflammatory substance (Smithson and Kawaja, 2010). In the study, ANXA3 was co-localized with markers of microglia in the CCI-induced neuropathic pain model in animals, which was consistent with Popa-Wagner's study (Junker et al., 2007). A growing body of evidence indicates that microglia in the spinal cord are important in the evolution and support of neuropathic pain (Taves et al., 2013; Piotrowska et al., 2016). Thus, microglia have received much attention as a key player in the mechanisms of neuropathic pain and as potential therapeutic targets (Taves et al., 2013; Ji et al., 2016). In this study, locomotor function was found intact using an automated four lane Rota-Rod (Rat Rota-Rod, Model 7750; Ugo Basile) in the rats with LV-shANXA3 and LV-NC injection and there was no significant difference among the LV-shANXA3, LV-NC, and NS-treated rats (data not shown).

The participation of crosstalk between microglia and neurons has been determined in the development of chronic pain, modulation of microglia reactivity from pro- to anti-inflammatory phenotypes could be a potential target for pain therapy (Ji et al., 2016). Previous studies has revealed that Annexin A2 regulates TRPA1-dependent nociception (Avenali et al., 2014) and may have an important role in neuropathic pain after peripheral nerve injury (Yamanaka et al., 2016). In our study, the data showed that ANXA3 expression is co-localized with microglia and increased in the CCI-induced neuropathic pain model of rats. A developing body of proof indicates that spinal inflammatory processes are present in the development and maintenance of neuropathic pain (Schafer et al., 2014), which indicates that ANXA3 may play a role through microglia activation. Therefore, ANXA3 maybe be the target of neuropathic pain treatments. Further exploration would be required to elucidate the precise role of ANXA3 in neuropathic pain.

In conclusion, the study indicate that ANXA3 may play an important role in CCI-induced neuropathic pain.

## Author contributions

WZ conceived and designed this study. WZ and WX conducted the behavioral tests. WX performed intrathecal catheter placement and staining experiments. ZS and TZ conducted PCR and western blot tests. WZ conducted statistical analysis. WZ organized the figures. WZ wrote the manuscript. WZ, XZ, and QG revised the manuscript. All authors reviewed the manuscript.

### Conflict of interest statement

The authors declare that the research was conducted in the absence of any commercial or financial relationships that could be construed as a potential conflict of interest.
