# Supplementary material for: Proteomic Identification of an Upregulated Isoform of Annexin A3 in the Spinal Cords of Rats in a Neuropathic Pain Model
Source: Front Neurosci. 2017 Sep 5;11:484. doi: 10.3389/fnins.2017.00484 (PMC5591859; doi:10.3389/fnins.2017.00484)
Supplement: Supplementary file 1 [file Presentation1.pdf]

Supplementary Information for

Proteomic identification of an Upregulated Isoform of Annexin A3 in the Spinal Cords of Rats in a Neuropathic Pain Model

Wangyuan Zou[1]*, Wei Xu [1], Zongbin Song[1], Tao Zhong[1], Yingqi Weng[1], Changsheng Huang[1], Maoyu Li[2], Chuanlei Zhang[1], Xianquan Zhan [2, 3], Qulian Guo[1]*

1. Department of Anesthesiology, Xiangya Hospital, Central South University, Changsha, Hunan 410008, China
2. Key Laboratory of Cancer Proteomics of Chinese Ministry of Health, Xiangya Hospital, Central South University, Changsha, Hunan 410008, China
3. Hunan Engineering Laboratory for Structural Biology and Drug Design, Xiangya Hospital, Central South University, Changsha, Hunan, China.

[*] To whom correspondence should be addressed:
Department of Anesthesiology
Xiangya Hospital
Central South University
87 Xiangya Road
Changsha, Hunan, 410008
China
Tel: 86-731-84327413
Fax: 86-731-84327413
E-mail: wangyuanzou@csu.edu.cn or qulianguo@hotmail.com

A. MALDI-TOF MS spectrum (spot 1)

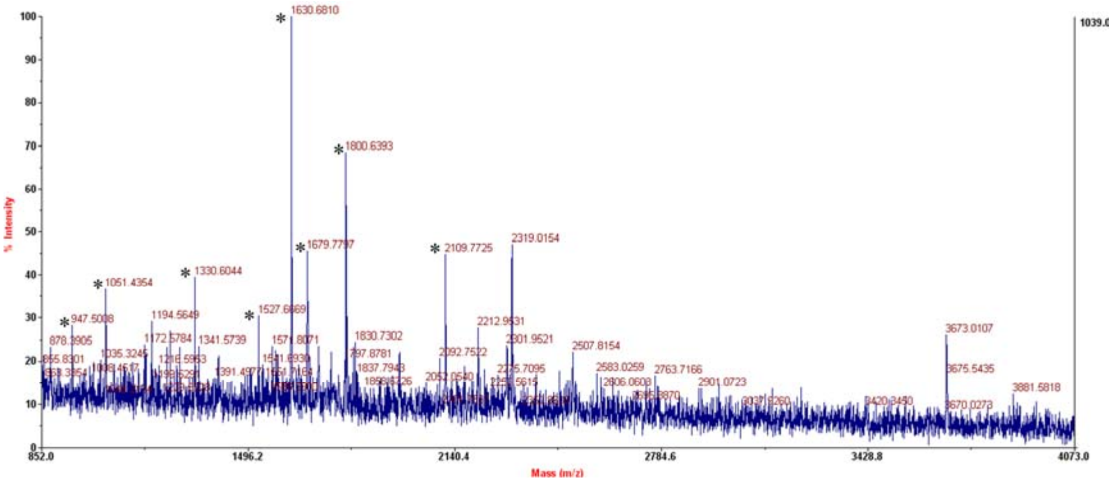

B. Peptides detected by MALDI-TOF-MS

Peptide	Amino acid sequence	$[M+H]^+$	Matched
46-53	MDELQLFR	1051.4330	+
46 - 60	MDELQLFRGDTVLLK	1777.7970	+
66 - 83	EAVCIVLSDDTCSDEKIR	2109.7740	+
148 - 155	KGDIFLVR	947.4930	+
324 - 338	IVSQLLTLMDGLKQR	1714.8550	+
454 - 465	WALSQSNPSALR	1329.6130	+
600 - 614	VINQILTEMDGMSTK	1679.7840	+
754 - 766	KYEMFAQTLQQSR	1629.6840	+
755 - 766	YEMFAQTLQQSR	1501.6090	+

C. Matched peptides (Bold Red) in the TERA amino acid sequence

1 MASGADSKGD DLSTAILKQK NRPNRLIVDE AINEDNSVVS LSQPK**MDELQ**
51 **LFRGDTVLLK** GKKRR**EAVCI VLSDDTCSDE KIR**MNRVVRN NLRVRLGDVI
101 SIQPCPDVKY GKRIHVLPID DTVEGITGNL FEVYLKPYFL EAYRPIR**KGD**
151 **IFLVR**GGMRA VEFKVVETDP SPYCIVAPDT VIHCEGEPIK REDEEESLNE
201 VGYDDIGGCR KQLAQIKEMV ELPLRHPALF KAIGVKPPRG ILLYGPPGTG
251 KTLIARAVAN ETGAFFFLIN GPEIMSKLAG ESESNLRKAF EEAEKNAPAI
301 IFIDELDAIA PKREKTHGEV ERR**IVSQLLT LMDGLKQR**AH VIVMAATNRP
351 NSIDPALRRF GRFDREVDIG IPDATGRLEI LQIHTKNMKL ADDVDLEQVA
401 NETHGHVGAD LAALCSEAAL QAIRKKMDLI DLEDETIDAE VMNSLAVTMD
451 DFR**WALSQSN PSALR**ETVVE VPQVTWEDIG GLEDVKRELQ ELVQYPVEHP
501 DKFLKFGMTP SKGVLFYGPP GCGKTLLAKA IANECQANFI SIKGPELLTM
551 WFGESEANVR EIFDKARQAA PCVLFFDELD SIAKARGGNI GDGGGAADR**V**
601 **INQILTEMDG MSTK**KNVFII GATNRPDIID PAILRPGRLD QLIYIPLPDE
651 KSRVAILKAN LRKSPVAKDV DLEFLAKMTN GFSGADLTEI CQRACKLAIR
701 ESIESEIRRE RERQTNPSAM EVEEDDPVPE IRRDHFEEAM RFARRSVSDN

751 DIR**KYEMFAQ TLQQSR**GFGS FRFPSGNQGG AGPSQGSGGG TGGNVYTEDN
801 DDDLYG

A. MALDI-TOF MS spectrum (spot 2)

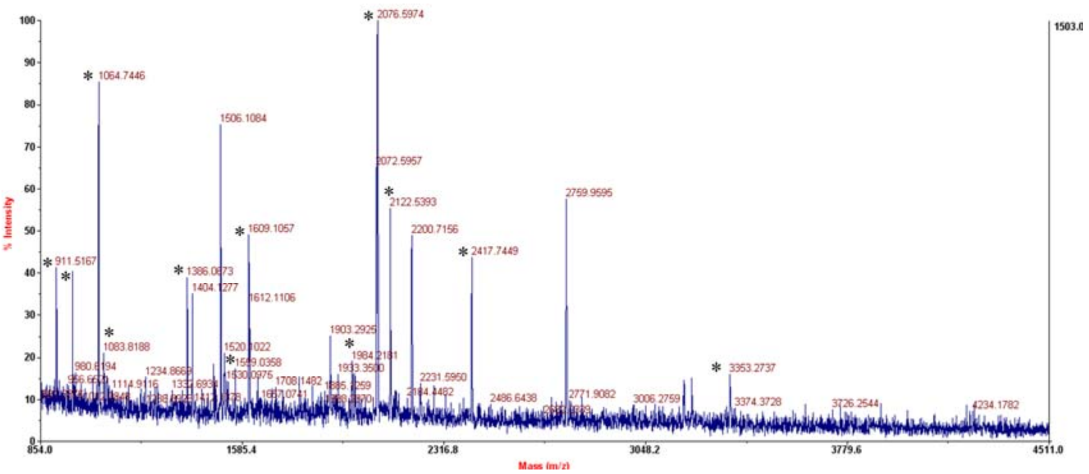

B. Peptides detected by MALDI-TOF-MS

Peptide	Amino acid sequence	$[M+H]^+$	Matched
63 - 68	FCYHER	911.5130	+
185 - 200	FASEIAGVDDLGTTGR	1609.1020	+
247 - 266	KTESIDVMDAVGSNIVVSTR	2121.4770	+
248 - 266	TESIDVMDAVGSNIVVSTR	1993.3830	+
292 - 299	FAYDGLKR	969.6620	+
409 - 417	FEAPLFNAR	1064.7480	+
429 - 450	VALIGSPVDLTYRYDHLGDSPK	2416.7160	+
451 - 464	ILQDIASGNHEFSK	1559.0540	+
465 - 483	VLNAAKKPMVVLGSSALQR	1982.3960	+
471 - 483	KPMVVLGSSALQR	1386.0600	+
519 - 538	IASQVAALDLGYKPGVEAIR	2071.5630	+
563 - 592	DCFIVYQGHHGDVGAPIADVILPGAAYTEK	3214.0580	+
646 - 655	LGEVSPNLVR	1083.8080	+
646 - 673	LGEVSPNLVRYDDVEEANYFQQASELAK	3185.0730	+
674 - 702	LVDQEFLADPLVPPQLTIKDFYMTDSISR	3352.2110	+

C. Matched peptides (Bold Red) in the NADH amino acid sequence

1 MLRIPVKRAL IGLSKSPKGY VRSTGTAASN LIEVFVDGQS VMVEPGTTVL
51 QACEKVGMQI PR**FCYHER**LS VAGNCRMCLV EIEKAPKVVA ACAMPVMKGW
101 NILTNSEKSK KAREGVMEFL LANHPLDCPI CDQGGECDLQ DQSMMFGSDR
151 SRFLEGKRAV EDKNIGPLVK TIMTRCIQCT RCIR**FASEIA GVDDLGTTGR**
201 GNDMQVGTYI EKMFMSELSG NIIDICPVGA LTSKPYAFTA RPWETR**KTES**
251 **IDVMDAVGSN IVVSTR**TGEV MRILPRMHED INEEWISDKT R**FAYDGLKR**Q
301 RLTEPMVRNE K**GLLTYTSWE DALSRVAGML QSFEGK**AVAA IAGGLVDAEA
351 LVALKDLLNK VDSDTLCTEE IFPNEGAGTD LRSNYLLNTT IAGVEEADVV
401 LLVGTNPR**FE APLFNAR**IRK SWLHNDLK**VA LIGSPVDLTY RYDHLGDSPK**

451 **ILQDIASGNH EFSKVLNAAK KPMVVLGSSA LQR**DDGAAIL AAVSSIAQKI

501 RVASGAAAEW KVMNILHR**IA SQVAALDLGY KPGVEAIR**KN PPKLLFLLGA

551 DGGCITRQDL PK**DCFIVYQG HHGDVGAPIA DVILPGAAYT EK**SATYVNTE

601 GRAQQTKVAV TPPGLAREDW KIIRALSEIA GITLPYDTLD QVRNR**LGEVS**

651 **PNLVRYDDVE EANYFQQASE LAKLVDQEFL ADPLVPPQLT IKDFYMTDSI**

701 **SR**ASQTMAKC VKAVTEGAQA VEEPSIC

A. MALDI-TOF MS spectrum (spot3)

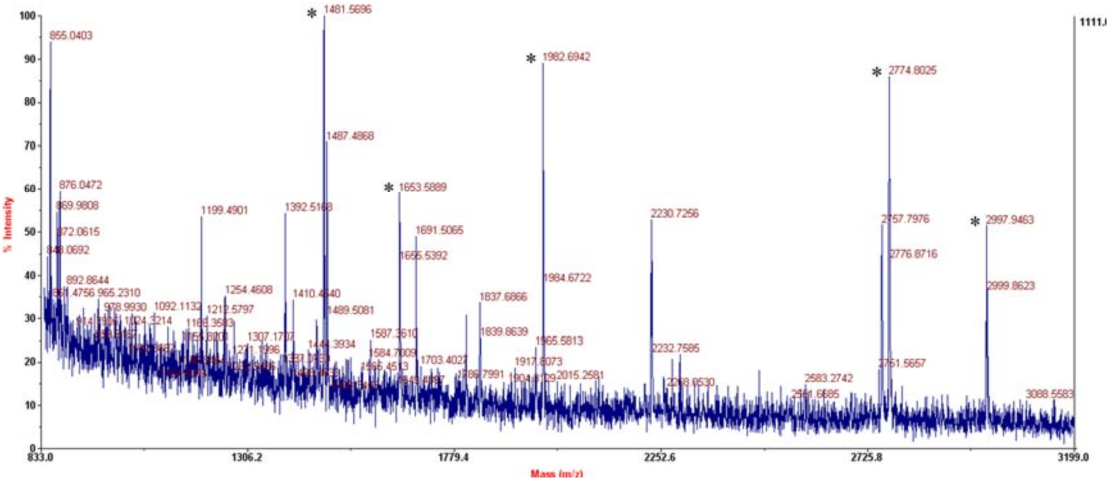

B. Peptides detected by MALDI-TOF-MS

Peptide	Amino acid sequence	$[M+H]^+$	Matched
4 - 25	GPAVGIDLGTTYSCVGVFQHGK	2262.6820	+
57 - 72	NQVAMNPTNTVFDAKR	1805.5470	+
89 - 102	HWPFMVVNDAGRPK	1653.5380	+
138 - 155	TVTNAVVTVPAYFNDSQR	1981.6220	+
273 - 299	TLSSSTQASIEIDSLYEGIDFYTSITR	2996.9690	+
300 - 311	ARFEELNADLFR	1480.4660	+
424 - 447	QTQTFTTYSDNQPGVLIQVYEGER	2773.8670	+

C. Matched peptides (Bold Red) in the HSC70 amino acid sequence

1 MSK**GPAVGID LGTTYSCVGV FQHGK**VEIIA NDQGNRTTPS YVAFTDTERL
51 IGDAAK**NQVA MNPTNTVFDA KR**LIGRRFDD AVVQSDMK**HW PFMVVNDAGR**
101 **PK**VQVEYKGE TKSFYPEEVS SMVLTKMKEI AEAYLGK**TVT NAVVTVPAYF**
151 **NDSQR**QATKD AGTIAGLNVL RIINEPTAAA IAYGLDKKVG AERNVLIFDL
201 GGGTFDVSIL TIEDGIFEVK STAGDTHLGG EDFDNRMVNH FIAEFKRKHK
251 KDISENKRAV RRLRTACERA KR**TLSSSTQA SIEIDSLYEG IDFYTSITRA**
301 **RFEELNADLF R**GTLDPVEKA LRDAKLDKSQ IHDIVLVGGS TRIPKIQKLL
351 QDFFNGKELN KSINPDEAVA YGAAVQAAIL SGDKSENVQD LLLLDVTPLS
401 LGIETAGGVM TVLIKRNTTI PTK**QTQTFTT YSDNQPGVLI QVYEGER**AMT
451 KDNNLLGKFE LTGIPPAPRG VPQIEVTFDI DANGILNVSA VDKSTGKENK
501 ITITNDKGRL SKEDIERMVQ EAEKYKAEDE KQRDKVSSKN SLESYAFNMK
551 ATVEDEKLQG KINDEDKQKI LDKCNEIISW LDKNQTAEKE EFEHQQKELE
601 KVCNPIITKL YQSAGGMPGG MPGGFPGGGA PPSGGASSGP TIEEVD

A. MALDI-TOF MS spectrum (spot 4)

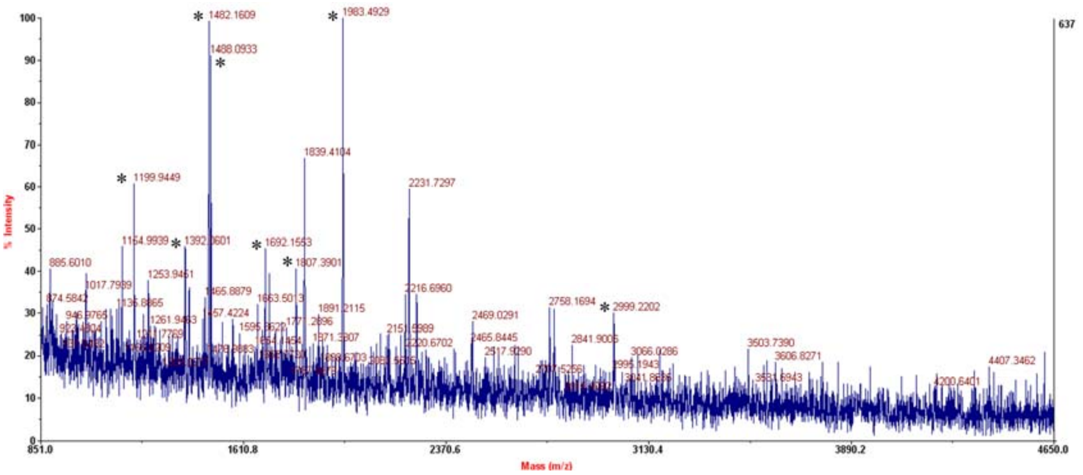

B. Peptides detected by MALDI-TOF-MS

Peptide	Amino acid sequence	$[M+H]^+$	Matched
37 - 49	TTPSYVAFTDTER	1488.0990	+
57 - 72	NQVAMNPTNTVFDAKR	1806.3720	+
138 - 155	TVTNAVVTVPAYFNDSQR	1982.5020	+
160 - 171	DAGTIAGLNVLR	1199.9640	+
221 - 236	STAGDTHLGGEDFDNR	1692.1830	+
237 - 247	MVNHFIAEFKR	1392.0750	+
273 - 299	TLSSSTQASIEIDSLYEGIDFYTSITR	2998.1880	+
300 - 311	ARFEELNADLFR	1481.1550	+
326 - 342	LDKSQIHDIVLVGGSTR	1838.4920	+
362 - 384	SINPDEAVAYGAAVQAAILSGDK	2260.7990	+
424 - 447	QTQTFTTYSDNQPGVLIQVYEGER	2775.0770	+

C. Matched peptides (Bold Red) in the HSC70 amino acid sequence

1 MSKGPAVGID LGTTYSCVGV FQHGKVEIIA NDQGNR**TTPS YVAFTDTER**L
51 IGDAAK**NQVA MNPTNTVFDA KR**LIGRRFDD AVVQSDMKHW PFMVVNDAGR
101 PKVQVEYKGE TKSFYPEEVS SMVLTKMKEI AEAYLGK**TVT NAVVTVPAYF**
151 **NDSQR**QATK**D AGTIAGLNVL R**IINEPTAAA IAYGLDKKVG AERNVLIFDL
201 GGGTFDVSIL TIEDGIFEVK **STAGDTHLGG EDFDNRMVNH FIAEFKR**KHK
251 KDISENKRAV RRLRTACERA KR**TLSSSTQA SIEIDSLYEG IDFYTSITRA**
301 **RFEELNADLF R**GTLDPVEKA LRDAK**LDKSQ IHDIVLVGGS TR**IPKIQKLL
351 QDFFNGKELN K**SINPDEAVA YGAAVQAAIL SGDK**SENVQD LLLLDVTPLS
401 LGIETAGGVM TVLIKRNTTI PTK**QTQTFTT YSDNQPGVLI QVYEGER**AMT
451 KDNNLLGKFE LTGIPPAPRG VPQIEVTFDI DANGILNVSA VDKSTGKENK
501 ITITNDKGRL SKEDIERMVQ EAEKYKAEDE KQRDKVSSKN SLESYAFNMK
551 ATVEDEKLQG KINDEDKQKI LDKCNEIISW LDKNQTAEKE EFEHQQKELE
601 KVCNPIITKL YQSAGGMPGG MPGGFPGGGA PPSGGASSGP TIEEVD

A. MALDI-TOF MS spectrum (spot 5)

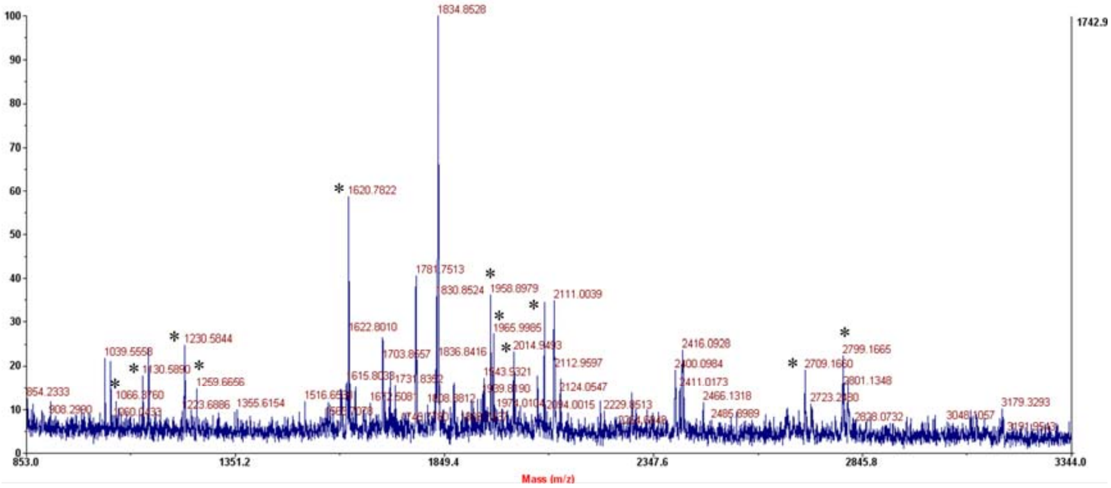

B. Peptides detected by MALDI-TOF-MS

Peptide	Amino acid sequence	$[M+H]^+$	Matched
1 - 19	MREIVHIQAGQCGNQIGAK	2109.9740	+
47 - 62	INVYYNEAAGNKYVPR	1870.8500	+
78 - 103	SGPFGQIFRPDNFVFGQSGAGNNWAK	2798.1530	+
104 - 121	GHYTEGAELVDSVLDVVR	1958.9080	+
217 - 241	LTTPTYGDLNHLVSATMSGVTTCLR	2708.1630	+
242 - 251	FPGQLNADLR	1130.5870	+
242 - 252	FPGQLNADLRK	1258.6740	+
253 - 262	LAVNMVPFPR	1143.6220	+
263 - 276	LHFFMPGFAPLTSR	1620.8000	+
263 - 276	LHFFMPGFAPLTSR	1636.7920	+
310 - 318	YLTVAAIFR	1053.5920	+
363 - 380	MSATFIGNSTAIQELFKR	2013.9550	+
381 - 390	ISEQFTAMFR	1229.6140	+

C. Matched peptides (Bold Red) in the Tubulin amino acid sequence

1 **MREIVHIQAG QCGNQIGAK**F WEVISDEHGI DPTGSYHGDS DLQLER**INVY**
51 **YNEAAGNKYV PR**AILVDLEP GTMDSVR**SGP FGQIFRPDNF VFGQSGAGNN**
101 **WAKGHYTEGA ELVDSVLDVV RK**ESESCDCL QGFQLTHSLG GGTGSGMGTL
151 LISKIREEYP DRIMNTFSVM PSPKVSDTVV EPYNATLSVH QLVENTDETY
201 SIDNEALYDI CFRTLK**LTTP TYGDLNHLVS ATMSGVTTCL RFPGQLNADL**
251 **RKLAVNMVPF PRLHFFMPGF APLTSR**GSQQ YRALTVPELT QQMFDSKNMM
301 AACDPRHGR**Y LTVAAIFR**GR MSMKEVDEQM LNVQNKNSSY FVEWIPNNVK
351 TAVCDIPPRG LK**MSATFIGN STAIQELFKR ISEQFTAMFR** RKAFLHWYTG
401 EGMDEMEFTE AESNMNDLVS EYQQYQDATA DEQGEFEEEE GEDEA

A. MALDI-TOF MS spectrum (spot 6)

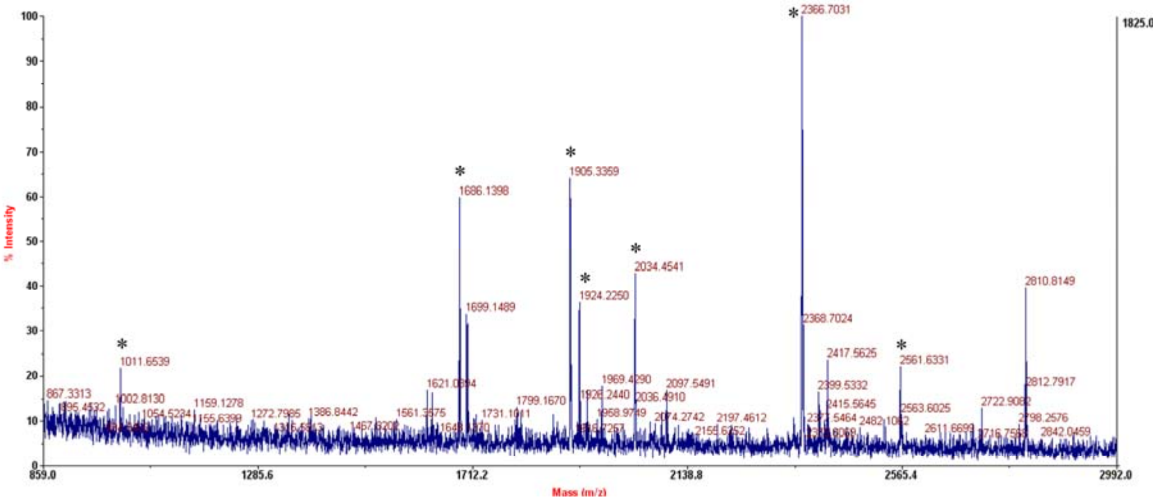

B. Peptides detected by MALDI-TOF-MS

Peptide	Amino acid sequence	$[M+H]^+$	Matched
97 - 121	LVQDVANNTNEEAGDGTTTATVLAR	2560.6330	+
134 - 142	GANPVEIRR	1011.6500	+
206 - 221	TLNDELEIIEGMKFDR	1923.2330	+
206 - 221	TLNDELEIIEGMKFDR	1939.2330	+
250 - 268	KISSVQSIVPALEIANAHR	2033.4540	+
251 - 268	ISSVQSIVPALEIANAHR	1905.3260	+
269 - 290	KPLVIIAEDVDGEALSTLVLNR	2365.7010	+
345 - 359	VGEVIVTKDDAMLLK	1631.1390	+
430 - 446	AAVEEGIVLGGGCALLR	1685.1500	+

C. Matched peptides (Bold Red) in the HSP60 amino acid sequence

1 MLRLPTVLRQ MRPVSRALAP HLTRAYAKDV KFGADARALM LQGVDLLADA
51 VAVTMGPKGR TVIIEQSWGS PKVTKDGVTV AKSIDLKDKY KNIGAK**LVQD**
101 **VANNTNEEAG DGTTTATVLA R**SIAKEGFEK ISK**GANPVEI RR**GVMLAVDA
151 VIAELKKQSK PVTTPEEIAQ VATISANGDK DIGNIISDAM KKVGRKGVIT
201 VKDGK**TLNDE LEIIEGMKFD R**GYISPYFIN TSKGQKCEFQ DAYVLLSEK**K**
251 **ISSVQSIVPA LEIANAHRKP LVIIAEDVDG EALSTLVLNR** LKVGLQVVAV
301 KAPGFGDNRK NQLKDMAIAT GGAVFGEEGL NLNLEDVQAH DLGK**VGEVIV**
351 **TKDDAMLLK**G KGDKAHIEKR IQEITEQLDI TTSEYEKEKL NERLAKLSDG
401 VAVLKVGGTS DVEVNEKKDR VTDALNATR**A AVEEGIVLGG GCALLR**CIPA
451 LDSLKPANED QKIGIEIIKR ALKIPAMTIA KNAGVEGSLI VEKILQSSSE
501 VGYDAMLGDF VNMVEKGIID PTKVVRTALL DAAGVASLLT TAEAVVTEIP
551 KEEKDPGMGA MGGMGGGMGG GMF

A. MALDI-TOF MS spectrum (spot 7)

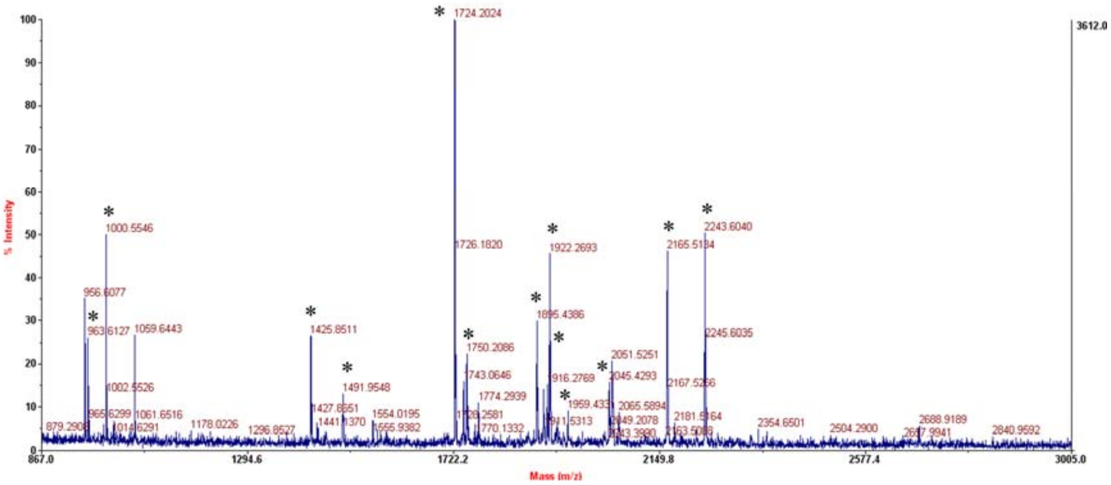

B. Peptides detected by MALDI-TOF-MS

Peptide	Amino acid sequence	$[M+H]^+$	Matched
69-76	MVEGFFDR	1000.5540	+
108 - 123	IIKPCNHVLSLSFPIR	1894.4380	+
125 - 136	DDGSWEVIEGYR	1425.8600	+
137 - 143	AQHSQHR	863.4720	+
212 - 231	KGFIGPGIDVPAPDMSTGER	2044.4190	+
213 - 231	GFIGPGIDVPAPDMSTGER	1916.2980	+
303 - 318	TFVVQGFGNVGLHSMR	1749.2120	+
347 - 363	ELEDFKLQHGSILGFPK	1958.3770	+
400 - 420	IIAEGANGPTTPEADKIFLER	2242.5740	+
445 - 453	NLNHVSYGR	1059.6440	+
454 - 460	LTFKYER	956.6030	+
461 - 476	DSNYHLLMSVQESLER	1921.2680	+
481 - 496	HGGTIPVVPTAEFQDR	1724.1860	+
497 - 516	ISGASEKDIVHSGLAYTMER	2164.4730	+
504 - 516	DIVHSGLAYTMER	1491.9690	+
528 - 535	YNLGLDLR	963.6170	+

C. Matched peptides (Bold) in the GDH amino acid sequence

**1**	MYRRLGEVLL	LSRAGPAALG	SAAADSAALL	GWARGQPSAV	PQPGLTPVAR
**51**	RHYSEAATDR	EDDPNFFK**MV**	**EGFFDR**GASI	VEDKLVEDLK	TRENEEQKRN
**101**	RVRGILR**IIK**	**PCNHVLSLSF**	**PIRRDDGSWE**	**VIEGYRAQHS**	**QHR**TPCKGGI
**151**	RYSTDVSVDE	VKALASLMTY	KCAVVDVPFG	GAKAGVKINP	KNYTDNELEK
**201**	ITRRFTMELA	K**KGFIGPGID**	**VPAPDMSTGE**	**REMSWIADTY**	**ASTIGHYDIN**
**251**	**AHACVTGKPI**	**SQGGIHGR**IS	ATGRGVFHGI	ENFINEASYM	SILGMTPGLG
**301**	DK**TFVVQGFG**	**NVGLHSMR**YL	HRFGAKCVGV	GESDGSIWNP	DGIDPK**ELED**
**351**	**FKLQHGSILG**	**FPK**AKVYEGS	**ILEADCDILI**	**PAASEK**QLTK	SNAPRVKAK**I**

401 IAEGANGPTT PEADKIFLER NIMVIPDLYL NAGGVTVSYF EWLK**NLNHVS**
451 YGRLTFKYER DSNYHLLMSV QESLERKFGK **HGGTIPVVPT AEFQDRISGA**
501 SEKDIVHSGL AYTMERSARQ IMRTAMK**YNL GLDLRTAAYV NAIEKVFK**VY
551 NEAGVTFT

A. MALDI-TOF MS spectrum (spot 8)

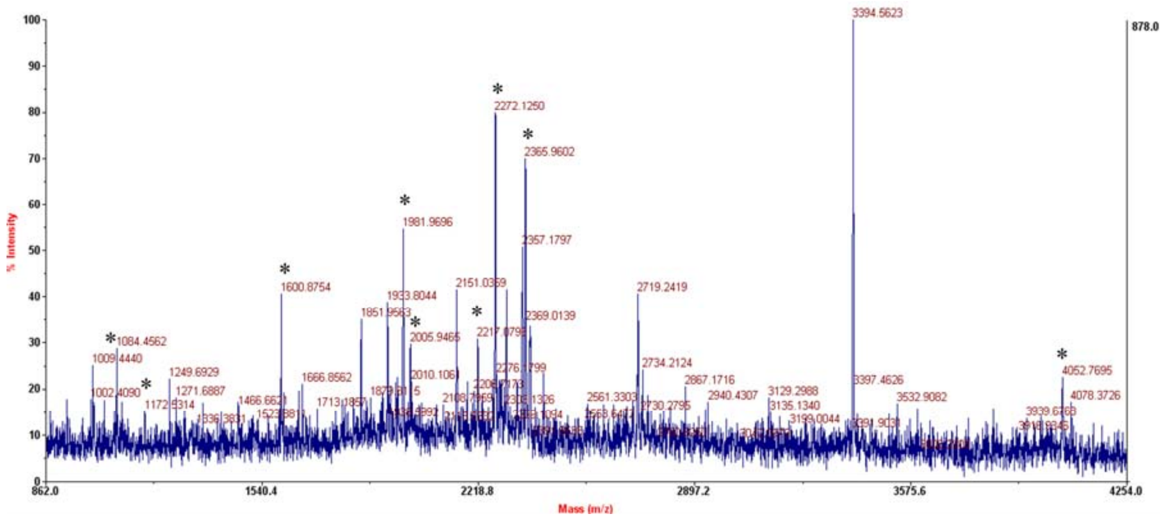

B. Peptides detected by MALDI-TOF-MS

Peptide	Amino acid sequence	$[M+H]^+$	Matched
83 - 97	ASLQNLLSASQAQLR	1599.8770	+
106-142	AFTSQASATMHLPATIGDYTDFYSSLQHATNVGIMFR	4049.7500	+
143-162	GKENALLPNWLHLPVGYHGR	2271.2040	+
163-174	ASSVVVSGTPIR	1172.5610	+
175-194	RPMGQMRPDNSKPPVYGASK	2216.0580	+
195-211	RLDMELEMAFFVGPGNR	1981.9410	+
221-237	AQEHIFGMVLMNDWSAR	2004.9210	+
372-381	AIDVGQGQTR	1044.4740	+
382-402	TFLLDGDEVIITGHCQGDGYR	2366.0740	+

C. Matched peptides (Bold Red) in the Fumarylacetoacetase amino acid sequence

1 MSFIPVAEDS DFPIQNLPYG VFSTQSNPKP RIGVAIGDQI LDLSVIKHLF
51 TGPVLSKHQH VFDETTLNSF MGLGQAAWKE AR**ASLQNLLS ASQAQLR**DDK
101 ELRQR**AFTSQ ASATMHLPAT IGDYTDFYSS LQHATNVGIM FRGKENALLP**
151 **NWLHLPVGYH GRASSVVVSG TPIRRPMGQM RPDNSKPPVY GASKRLDMEL**
201 **EMAFFVGPGN R**FGEPIPISK **AQEHIFGMVL MNDWSAR**DIQ QWEYVPLGPF
251 LGKSFGTTIS PWVVPMDALM PFVVPNPKQD PKPLPYLCHS QPYTFDINLS
301 VALKGEGMSQ AATICRSNFK HMYWTILQQL THHSVNGCNL RPGDLLASGT
351 ISGSDPESFG SMLELSWKGT K**AIDVGQGQT RTFLLDGDEV IITGHCQGDG**
401 **YR**VGFGQCAG KVLPALSPA

A. MALDI-TOF MS spectrum (spot 9)

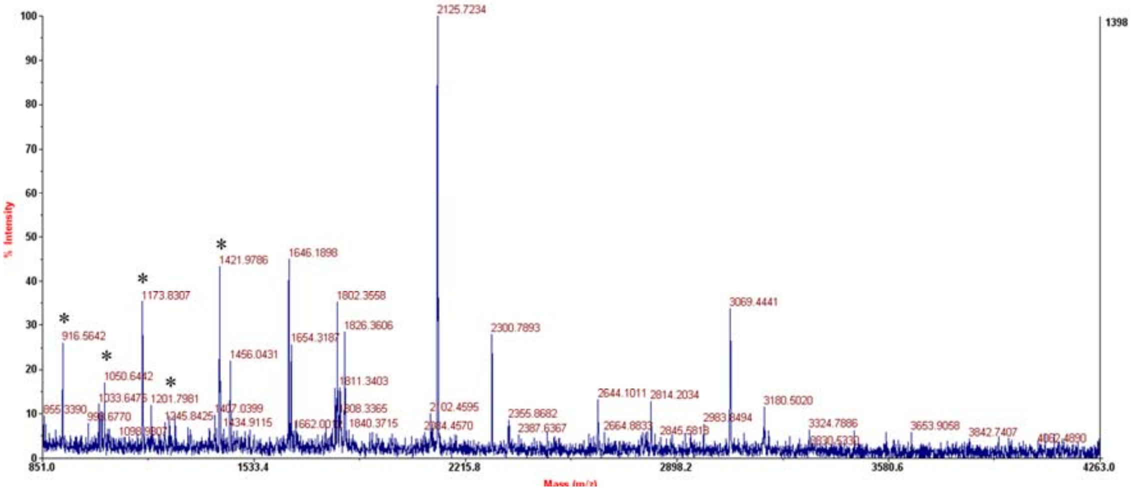

B. Peptides detected by MALDI-TOF-MS

Peptide	Amino acid sequence	$[M+H]^+$	Matched
15-22	ELSDIALR	916.5810	+
61-69	QVLFSADDR	1050.6480	+
61-71	QVLFSADDRVK	1277.9280	+
102-111	GILVGIKVDK	1041.7720	+
305-315	ALQASALSAWR	1173.8340	+
319-331	DNAGAATEEFIKR	1421.9770	+
331-342	RAEMNGLAAQGK	1245.8630	+

C. Matched peptides (Bold Red) in the Fructose-bisphosphate aldolase C amino acid sequence

1 MPHSYPALSA EQKK**ELSDIA LR**IVAPGKGI LAADESVGSM AKRLSQIGVE
51 NTEENRRLYR **QVLFSADDRV K**KCIGGVIFF HETLYQKDDN GVPFVRTIQE
101 K**GILVGIKVD K**GVVPLAGTD GETTTQGLDG LLERCAQYKK DGADFAKWRC
151 VLKISDRTPS ALAILENANV LARYASICQQ NGIVPIVEPE ILPDGDHDLK
201 RCQFVTEKVL AAVYKALSDH HVYLEGTLLK PNMVTPGHAC PIKYSPEEIA
251 MATVTALRRT VPPAVPGVTF LSGGQSEEEA SLNLNAINRC SLPRPWALTF
301 SYGR**ALQASA LSAWR**GQR**DN AGAATEEFIK RAEMNGLAAQ GK**YEGSGDGG
351 AAAQSLYVAN HAY

A. MALDI-TOF MS spectrum (spot10)

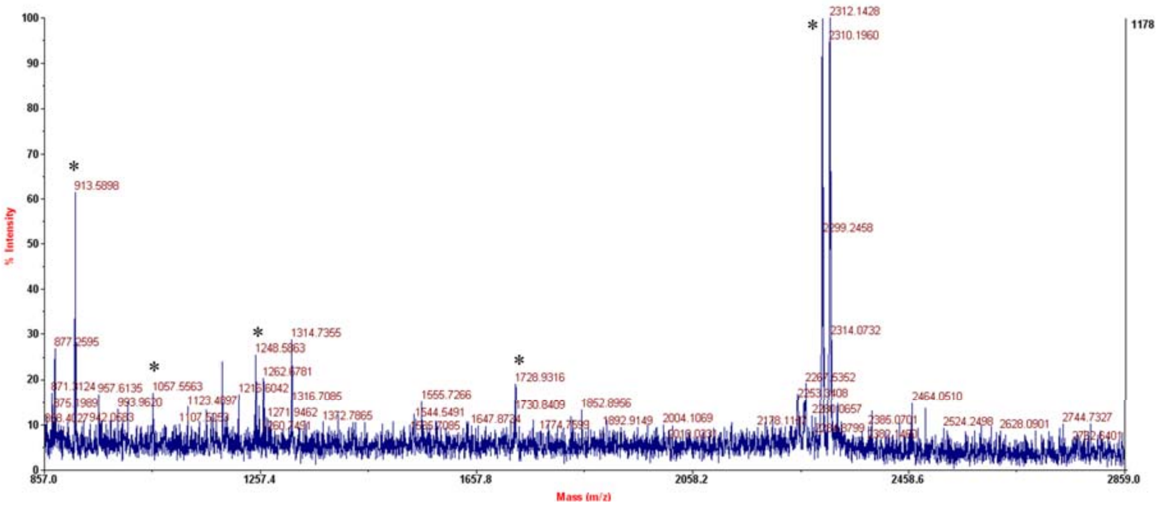

B. Peptides detected by MALDI-TOF-MS

Peptide	Amino acid sequence	$[M+H]^+$	Matched
92-100	IVVVTAGVR	913.5910	+
92-107	IVVVTAGVRQQEGESR	1727.8920	+
159-170	VIGSGCNLDSAR	1248.5780	+
171-178	FRYLMAEK	1057.5650	+
280-299	GMYGIENEVFLSLPCILNAR	2296.1350	+
309-318	LKDDEVAQLR	1186.6320	+

C. Matched peptides (Bold Red) in the LDH amino acid sequence

1 MATLKEKLIA PVADDETAVP NNKITVVGVG QVGMACAISI LGKSLADELA
51 LVDVLEDKLK GEMMDLQHGS LFLQTPKIVA DKDYSVTANS K**IVVVTAGVR**
101 **QQEGESR**LNL VQRNVNVFKF IIPQIVKYSP DCTIIVVSNP VDILTYVTWK
151 LSGLPKHR**VI GSGCNLDSAR FRYLMAEK**LG IHPSSCHGWI LGEHGDSSVA
201 VWSGVNVAGV SLQELNPEMG TDNDSENWKE VHKMVVDSAY EVIKLKGYTN
251 WAIGLSVADL IESMLKNLSR IHPVSTMVK**G MYGIENEVFL SLPCILNAR**G
301 LTSVINQK**LK DDEVAQLR**KS ADTLWDIQKD LKDL

A. MALDI-TOF MS spectrum (spot 11)

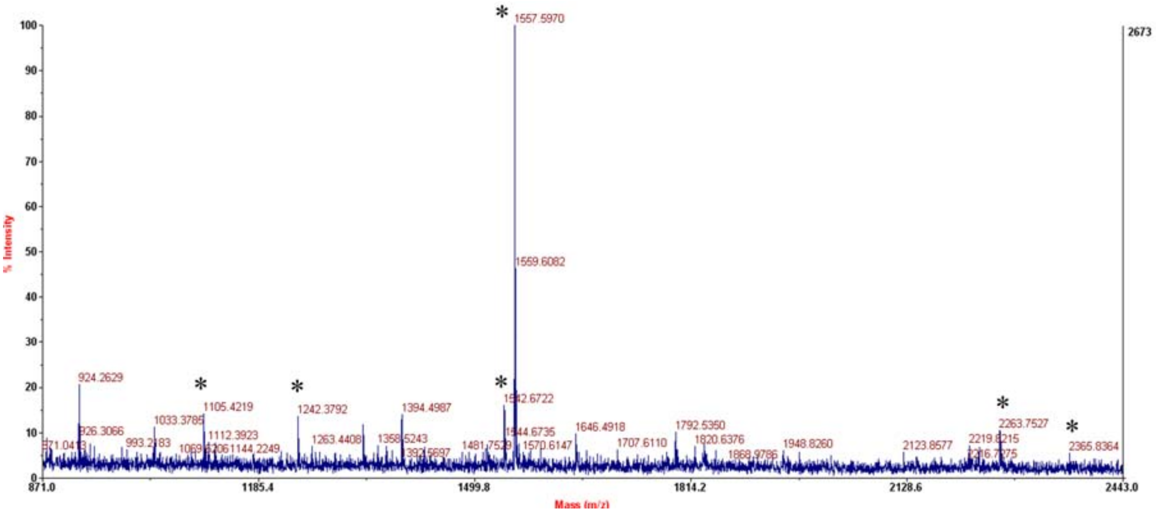

B. Peptides detected by MALDI-TOF-MS

Peptide	Amino acid sequence	$[M+H]^+$	Matched
70-78	RQDLFIVSK	1105.4340	+
156-169	AIGVSNFNPLQIER	1557.5990	+
244-256	TTAQVLIRFPIQR	1542.6660	+
276-294	FRYLMAEK	2263.7400	+
280-299	VFDFELSNEDMATLLSYNR	2296.1350	+
307-316	HKDYPFHAEV	1242.3780	+

C. Matched peptides (Bold Red) in the AR amino acid sequence

1 MASHLELNNG TKMPTLGLGT WKSPPGQVTE AVKVAIDMGY RHIDCAQVYQ
51 NEKEVGVALQ EKLKEQVVK**R QDLFIVSK**LW CTFHDQSMVK GACQKTLSDL
101 QLDYLDLYLI HWPTGFKPGP DYFPLDASGN VIPSDTDFVD TWTAMEQLVD
151 EGLVK**AIGVS NFNPLQIER**I LNKPGLKYKP AVNQIECHPY LTQEKLIEYC
201 HCKGIVVTAY SPLGSPDRPW AKPEDPSLLE DPRIKEIAAK YNK**TTAQVLI**
251 **RFPIQR**NLVV IPKSVTPARI AENFK**VFDFE LSNEDMATLL SYNR**NWRVCA
301 LMSCAK**HKDY PFHAEV**

A. MALDI-TOF MS spectrum (spot 12)

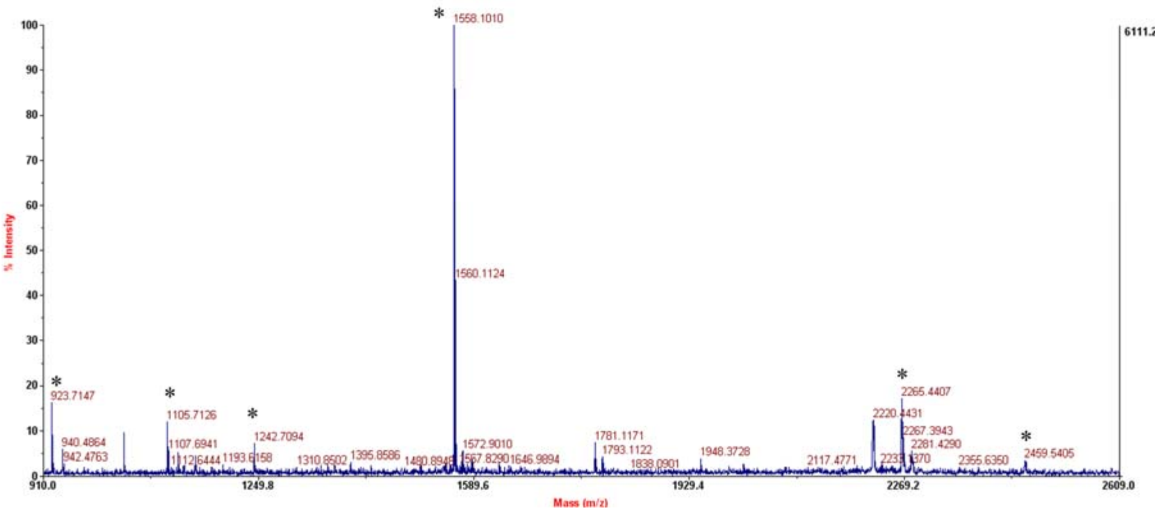

B. Peptides detected by MALDI-TOF-MS

Peptide	Amino acid sequence	$[M+H]^+$	Matched
34-41	VAIDMGYR	924.4990	+
42-62	HIDCAQVYQNEKEVGVALQEK	2458.5160	+
70-78	RQDLFIVSK	1105.7080	+
156-169	AIGVSNFNPLQIER	1558.0120	+
196-203	LIEYCHCK	1122.5820	+
244-251	TTAQVLIR	901.5900	+
276-294	VFDFELSNEDMATLLSYNR	2264.3420	+
307-316	HKDYPFHAEV	1242.7110	+

C. Matched peptides (Bold Red) in the AR amino acid sequence

1 MASHLELNNG TKMPTLGLGT WKSPPGQVTE AVK**VAIDMGY RHIDCAQVYQ**
51 **NEKEVGVALQ EK**LKEQVVK**R QDLFIVSK**LW CTFHDQSMVK GACQKTLSDL
101 QLDYLDLYLI HWPTGFKPGP DYFPLDASGN VIPSDTDFVD TWTAMEQLVD
151 EGLVK**AIGVS NFNPLQIER**I LNKPGLKYKP AVNQIECHPY LTQEK**LIEYC**
201 **HCK**GIVVTAY SPLGSPDRPW AKPEDPSLLE DPRIKEIAAK YNK**TTAQVLI**
251 **R**FPIQRNLVV IPKSVTPARI AENFK**VFDFE LSNEDMATLL SYNR**NWRVCA
301 LMSCAK**HKDY PFHAEV**

A. MALDI-TOF MS spectrum (spot 13)

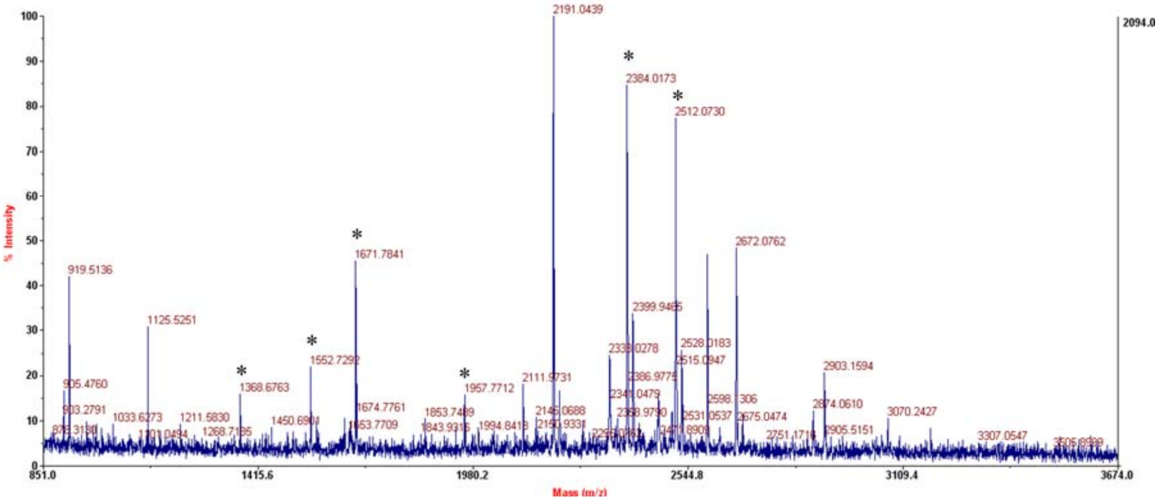

B. Peptides detected by MALDI-TOF-MS

Peptide	Amino acid sequence	[M+H]$^+$	Matched
9-16	RVLVYGGR	924.4990	+
53 -70	MTDSFTEQADQVTAEVGK	1956.7540	+
136-151	AALDGTPGMIGYGMAK	1552.7290	+
152-164	GAVHQLCQSLAGK	1368.6720	+
165-188	NSGMPSGAAAIAVLPVTLDTPMNR	2382.9970	+
165-189	NSGMPSGAAAIAVLPVTLDTPMNRK	2511.0690	+
218-233	RPNSGSLIQVVTTDGK	1671.8010	+

C. Matched peptides (Bold Red) in the DHPR amino acid sequence

1 MAASGEAR**RV LVYGGR**GALG SRCVQAFRAR NWWVASIDVV ENEEASASVI
51 VK**MTDSFTEQ ADQVTAEVGK** LLGDQKVDAI LCVAGGWAGG NAKSKSLFKN
101 CDLMWKQSIW TSTISSHLAT KHLKEGGLLT LAGAK**AALDG TPGMIGYGMA**
151 **KGAVHQLCQS LAGKNSGMPS GAAAIAVLPV TLDTPMNRK**S MPEADFSSWT
201 PLEFLVETFH DWITGNK**RPN SGSLIQVVTT DGK**TELTPAY F

A. MALDI-TOF MS spectrum (spot 14)

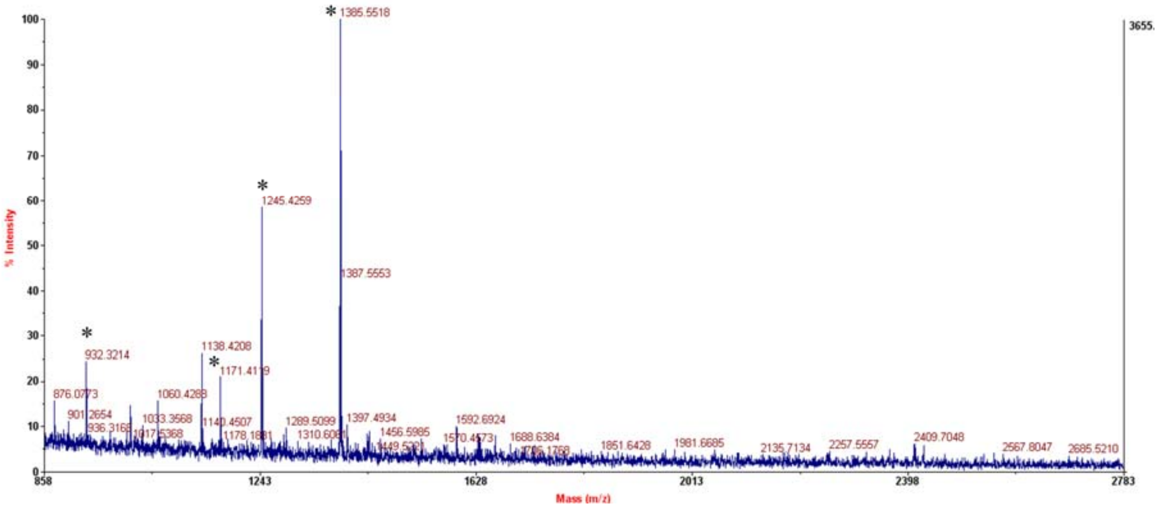

B. Peptides detected by MALDI-TOF-MS

Peptide	Amino acid sequence	$[M+H]^+$	Matched
2-13	PGKPVLHYFDGR	1385.6230	+
130-138	NRYFPAFEK	1171.5620	+
142-152	SHGQDYLVGNR	1245.5900	+
142-155	SHGQDYLVGNRLSR	1601.6710	+
197-204	FLQPGSQR	932.4410	+

C. Matched peptides (Bold Red) in the GSTA3 amino acid sequence

1 M**PGKPVLHYF DGR**GRMEPIR WLLAAAGVEF EEQFLKTRDD LARLRNDGSL
51 MFQQVPMVEI DGMKLVQTRA ILNYIATKYN LYGKDMKERA LIDMYAEGVA
101 DLDEIVLHYP YIPPGEKEAS LAKIKDKAR**N RYFPAFEK**VL K**SHGQDYLVG**
151 **NRLSR**ADVYL VQVLYHVEEL DPSALANFPL LKALRTRVSN LPTVKK**FLQP**
201 **GSQR**KPLEDE KCVESAVKIF S

A. MALDI-TOF MS spectrum (spot 15)

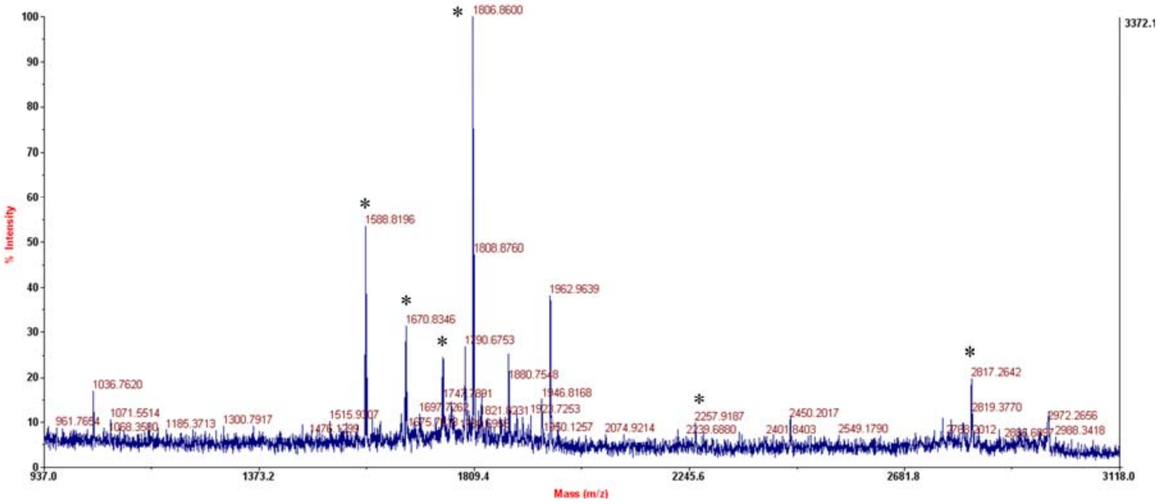

B. Peptides detected by MALDI-TOF-MS

Peptide	Amino acid sequence	$[M+H]^+$	Matched
32-45	MLQLVEESKDAGIR	1588.8210	+
46-59	TLVMLDEQGEQLER	1660.8090	+
104-119	AWGNNQDGVVASQPAR	1669.7700	+
120-135	VVDEREQMAISGGFIR	1806.8720	+
137-161	VTNDARENEMDENLEQVSGIIGNLR	2816.2520	+
162-176	HMALDMGNEIDTQNR	1744.7850	
162-180	HMALDMGNEIDTQNRQIDR	2256.9290	

C. Matched peptides (Bold Red) in the SNP25 amino acid sequence

1 MAEDADMRNE LEEMQRRADQ LADESLESTR R**MLQLVEESK DAGIRTLVML**
51 **DEQGEQLER**I EEGMDQINKD MKEAEKNLTD LGKFCGLCVC PCNKLKSSDA
101 YKK**AWGNNQD GVVASQPARV VDEREQMAIS GGFIR**R**VTND ARENEMDENL**
151 **EQVSGIIGNL RHMALDMGNE IDTQNRQIDR** IMEKADSNKT RIDEANQRAT
201 KMLGSG

A. MALDI-TOF MS spectrum (spot 16)

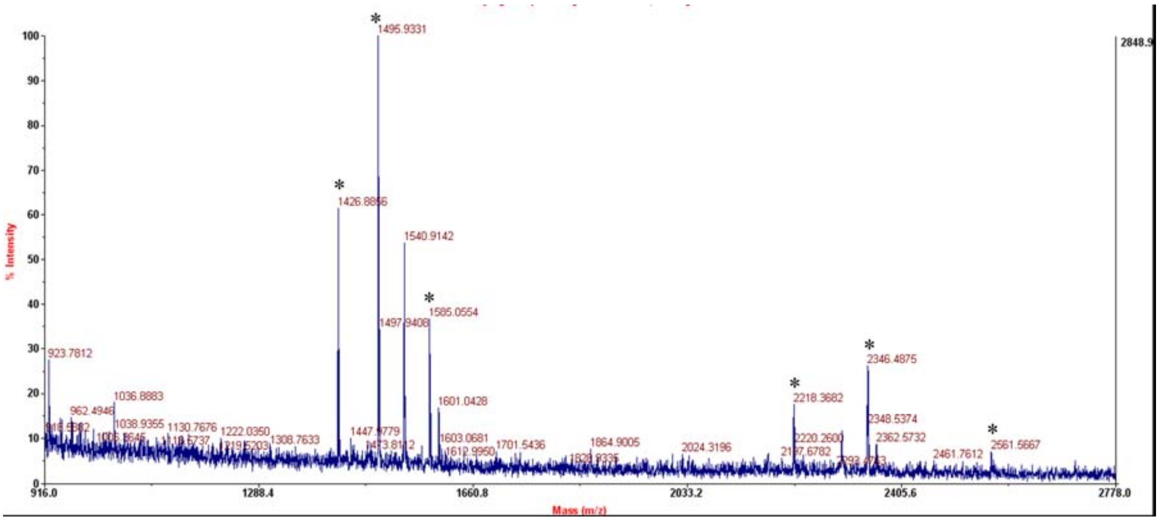

B. Peptides detected by MALDI-TOF-MS

Peptide	Amino acid sequence	$[M+H]^+$	Matched
32-44	YGYTHLSTGDLLR	1495.9390	+
64 -77	GELVPLETVLDMLR	1585.0450	+
108-128	KIAQPTLLLYVDAGPETMTQR	2345.4940	+
109-128	IAQPTLLLYVDAGPETMTQR	2217.3710	+
156-167	ATEPVISFYDKR	1425.8930	+
172-194	KVNAEGSVDTVFSQVCTYLDSLK	2560.5700	+

C. Matched peptides (Bold) in the AK amino acid sequence

1 MEDKLKKAKI IFVVGGPGSG KGTQCEKIVQ K**YGYTHLSTG DLLR**AEVSSG
51 SSRGKMLSSI MEK**GELVPLE TVLDMLR**DAM LAKVDSSNGF LIDGYPREVK
101 QGEEFER**KIA QPTLLLYVDA GPETMTQR**LL KRGETSGRVD DNEETIKKRL
151 ETYYK**ATEPV ISFYDKR**GIV R**KVNAEGSVD TVFSQVCTYL DSLK**

A. MALDI-TOF MS spectrum (spot 17)

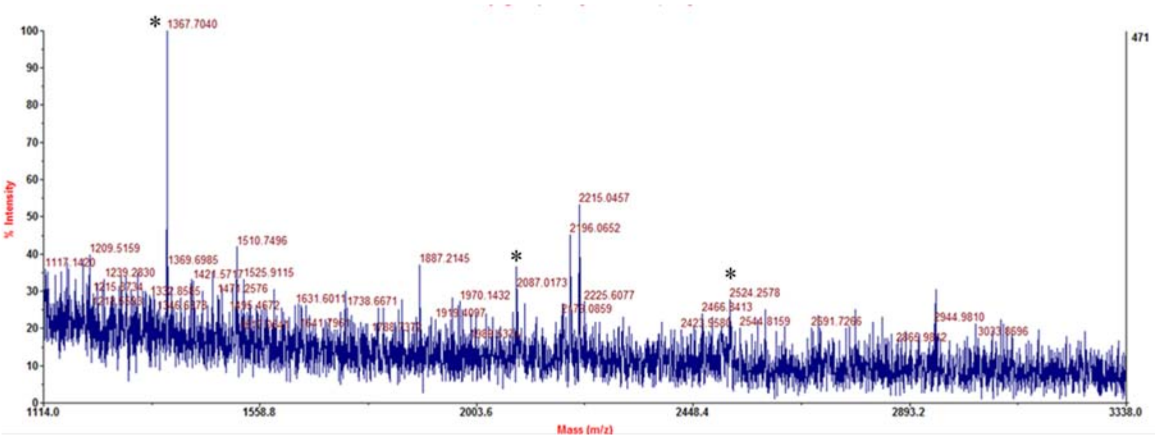

B. Peptides detected by MALDI-TOF-MS

Peptide	Amino acid sequence	$[M+H]^+$	Matched
72-92	HGGPADEERHVGDLGNVAAGK	2085.9560	+
93-116	DGVANVSIEDRVISLSGEHSIIGR	2523.1270	+
104-116	VISLSGEHSIIGR	1367.7090	+

C. Matched peptides (Bold) in the SOD amino acid sequence

1 MAMKAVCVLK GDGPVQGVIH FEQKASGEPV VVSGQITGLT EGEHGFHVHQ
51 YGDNTQGCTT AGPHFNPHSK K**HGGPADEER HVGDLGNVAA GKDGVANVSI**
101 **EDRVISLSGE HSIIGR**TMVV HEKQDDLGKG GNEESTKTGN AGSRLACGVI
151 GIAQ

A. MALDI-TOF MS spectrum (spot 18)

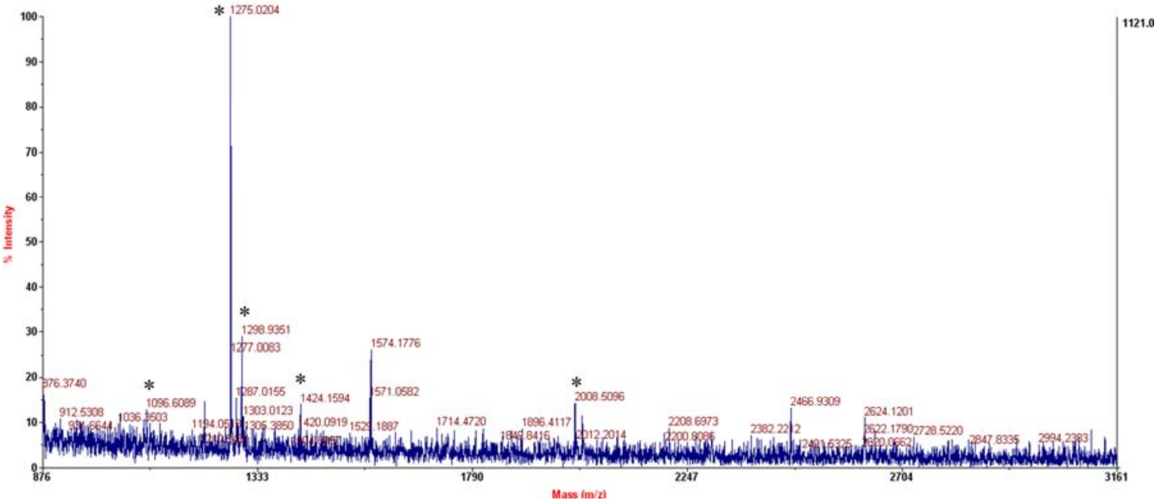

B. Peptides detected by MALDI-TOF-MS

Peptide	Amino acid sequence	$[M+H]^+$	Matched
19-31	VNPDDVGGEALGR	1298.9430	+
32-41	LLVVYPWTQR	1275.0190	+
42-60	YFDSFGDLSSASAIMGNPK	2007.5030	+
68 -77	VINAFNDGLK	1090.8120	
134-147	VVAGVASALAHKYH	1423.1460	

C. Matched peptides (Bold Red) in the Hemoglobin subunit beta-1 amino acid sequence

1 MVHLTDAEKA AVNGLWGK**VN PDDVGGEALG RLLVVYPWTQ RYFDSFGDLS**
51 **SASAIMGNPK** VKAHGKK**VIN AFNDGLKHLD NLK**GTFAHLS ELHCDKLHVD
101 PENFRLLGNM IVIVLGHHLG KEFTPCAQAA FQK**VVAGVAS ALAHKYH**

Supplementary Information for

Proteomic identification of an Upregulated Isoform of Annexin A3 in the Spinal Cords of Rats in a Neuropathic Pain Model

Wangyuan Zou[1]*, Wei Xu [1], Zongbin Song[1], Tao Zhong[1], Yingqi Weng[1], Changsheng Huang[1], Maoyu Li[2], Xianquan Zhan [2, 3], Qulian Guo[1]*

1. Department of Anesthesiology, Xiangya Hospital, Central South University, Changsha, Hunan 410008, China
2. Key Laboratory of Cancer Proteomics of Chinese Ministry of Health, Xiangya Hospital, Central South University, Changsha, Hunan 410008, China
3. Hunan Engineering Laboratory for Structural Biology and Drug Design, Xiangya Hospital, Central South University, Changsha, Hunan, China.

[*] To whom correspondence should be addressed:
Department of Anesthesiology
Xiangya Hospital
Central South University
87 Xiangya Road
Changsha, Hunan, 410008
China
Tel: 86-731-84327413
Fax: 86-731-84327413
E-mail: wangyuanzou@csu.edu.cn or qulianguo@hotmail.com

A. MALDI-TOF MS spectrum (spot 1)

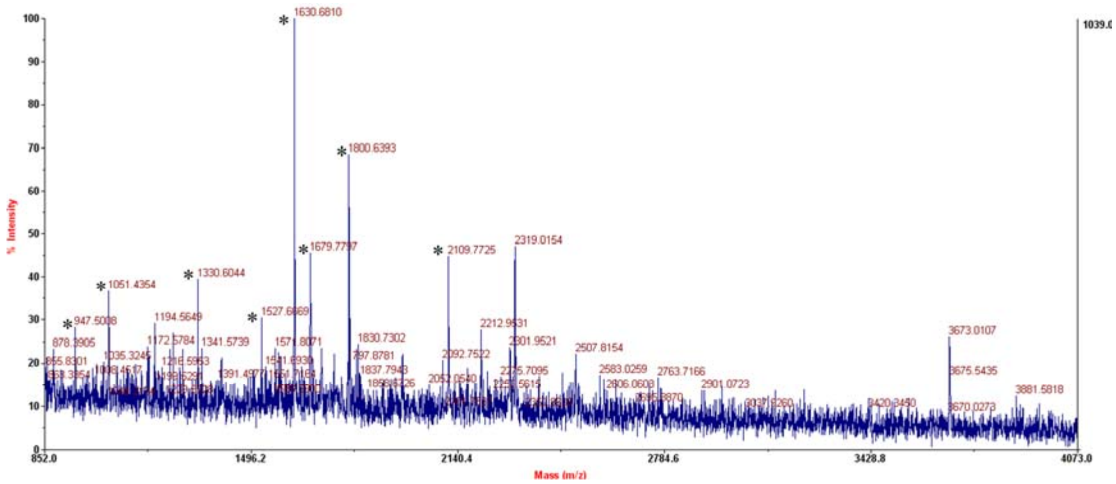

B. Peptides detected by MALDI-TOF-MS

Peptide	Amino acid sequence	$[M+H]^+$	Matched
46-53	MDELQLFR	1051.4330	+
46 - 60	MDELQLFRGDTVLLK	1777.7970	+
66 - 83	EAVCIVLSDDTCSDEKIR	2109.7740	+
148 - 155	KGDIFLVR	947.4930	+
324 - 338	IVSQLLTLMDGLKQR	1714.8550	+
454 - 465	WALSQSNPSALR	1329.6130	+
600 - 614	VINQILTEMDGMSTK	1679.7840	+
754 - 766	KYEMFAQTLQQSR	1629.6840	+
755 - 766	YEMFAQTLQQSR	1501.6090	+

C. Matched peptides (Bold Red) in the TERA amino acid sequence

1 MASGADSKGD DLSTAILKQK NRPNRLIVDE AINEDNSVVS LSQPK**MDELQ**
51 **LFRGDTVLLK** GKKRR**EAVCI VLSDDTCSDE KIR**MNRVVRN NLRVRLGDVI
101 SIQPCPDVKY GKRIHVLPID DTVEGITGNL FEVYLKPYFL EAYRPIR**KGD**
151 **IFLVR**GGMRA VEFKVVETDP SPYCIVAPDT VIHCEGEPIK REDEEESLNE
201 VGYDDIGGCR KQLAQIKEMV ELPLRHPALF KAIGVKPPRG ILLYGPPGTG
251 KTLIARAVAN ETGAFFFLIN GPEIMSKLAG ESESNLRKAF EEAEKNAPAI
301 IFIDELDAIA PKREKTHGEV ERR**IVSQLLT LMDGLKQR**AH VIVMAATNRP
351 NSIDPALRRF GRFDREVDIG IPDATGRLEI LQIHTKNMKL ADDVDLEQVA
401 NETHGHVGAD LAALCSEAAL QAIRKKMDLI DLEDETIDAE VMNSLAVTMD
451 DFR**WALSQSN PSALR**ETVVE VPQVTWEDIG GLEDVKRELQ ELVQYPVEHP
501 DKFLKFGMTP SKGVLFYGPP GCGKTLLAKA IANECQANFI SIKGPELLTM
551 WFGESEANVR EIFDKARQAA PCVLFFDELD SIAKARGGNI GDGGGAADR**V**
601 **INQILTEMDG MSTK**KNVFII GATNRPDIID PAILRPGRLD QLIYIPLPDE
651 KSRVAILKAN LRKSPVAKDV DLEFLAKMTN GFSGADLTEI CQRACKLAIR
701 ESIESEIRRE RERQTNPSAM EVEEDDPVPE IRRDHFEEAM RFARRSVSDN

751 DIR**KYEMFAQ TLQQSR**GFGS FRFPSGNQGG AGPSQGSGGG TGGNVYTEDN
801 DDDLYG

A. MALDI-TOF MS spectrum (spot 2)

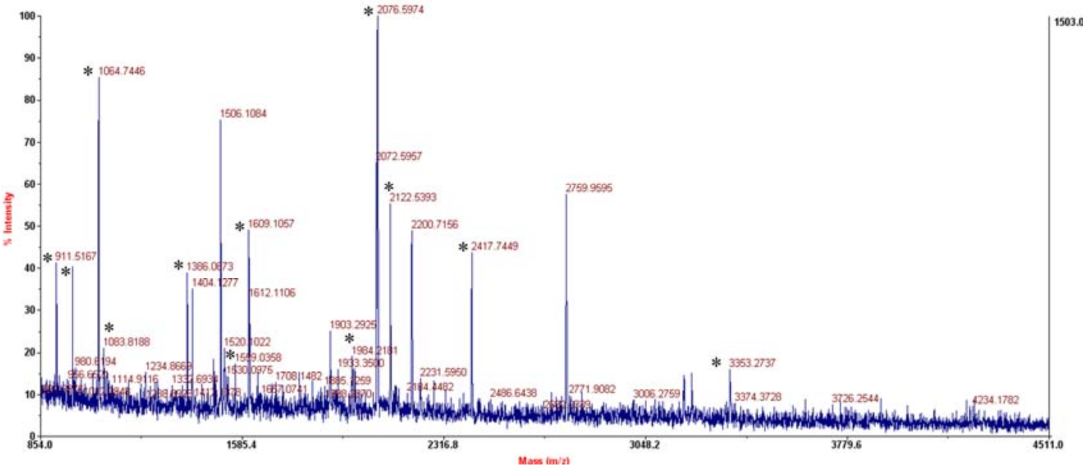

B. Peptides detected by MALDI-TOF-MS

Peptide	Amino acid sequence	$[M+H]^+$	Matched
63 - 68	FCYHER	911.5130	+
185 - 200	FASEIAGVDDLGTTGR	1609.1020	+
247 - 266	KTESIDVMDAVGSNIVVSTR	2121.4770	+
248 - 266	TESIDVMDAVGSNIVVSTR	1993.3830	+
292 - 299	FAYDGLKR	969.6620	+
409 - 417	FEAPLFNAR	1064.7480	+
429 - 450	VALIGSPVDLTYRYDHLGDSPK	2416.7160	+
451 - 464	ILQDIASGNHEFSK	1559.0540	+
465 - 483	VLNAAKKPMVVLGSSALQR	1982.3960	+
471 - 483	KPMVVLGSSALQR	1386.0600	+
519 - 538	IASQVAALDLGYKPGVEAIR	2071.5630	+
563 - 592	DCFIVYQGHHGDVGAPIADVILPGAAYTEK	3214.0580	+
646 - 655	LGEVSPNLVR	1083.8080	+
646 - 673	LGEVSPNLVRYDDVEEANYFQQASELAK	3185.0730	+
674 - 702	LVDQEFLADPLVPPQLTIKDFYMTDSISR	3352.2110	+

C. Matched peptides (Bold Red) in the NADH amino acid sequence

1 MLRIPVKRAL IGLSKSPKGY VRSTGTAASN LIEVFVDGQS VMVEPGTTVL
51 QACEKVGMQI PR**FCYHER**LS VAGNCRMCLV EIEKAPKVVA ACAMPVMKGW
101 NILTNSEKSK KAREGVMEFL LANHPLDCPI CDQGGECDLQ DQSMMFGSDR
151 SRFLEGKRAV EDKNIGPLVK TIMTRCIQCT RCIR**FASEIA GVDDLGTTGR**
201 GNDMQVGTYI EKMFMSELSG NIIDICPVGA LTSKPYAFTA RPWETR**KTES**
251 **IDVMDAVGSN IVVSTR**TGEV MRILPRMHED INEEWISDKT R**FAYDGLKR**Q
301 RLTEPMVRNE K**GLLTYTSWE DALSRVAGML QSFEGK**AVAA IAGGLVDAEA
351 LVALKDLLNK VDSDTLCTEE IFPNEGAGTD LRSNYLLNTT IAGVEEADVV
401 LLVGTNPR**FE APLFNAR**IRK SWLHNDLK**VA LIGSPVDLTY RYDHLGDSPK**

451 **ILQDIASGNH EFSKVLNAAK KPMVVLGSSA LQR**DDGAAIL AAVSSIAQKI
501 RVASGAAAEW KVMNILHR**IA SQVAALDLGY KPGVEAIR**KN PPKLLFLLGA
551 DGGCITRQDL PK**DCFIVYQG HHGDVGAPIA DVILPGAAYT EK**SATYVNTE
601 GRAQQTKVAV TPPGLAREDW KIIRALSEIA GITLPYDTLD QVRNR**LGEVS**
651 **PNLVRYDDVE EANYFQQASE LAKLVDQEFL ADPLVPPQLT IKDFYMTDSI**
701 **SR**ASQTMAKC VKAVTEGAQA VEEPSIC

A. MALDI-TOF MS spectrum (spot3)

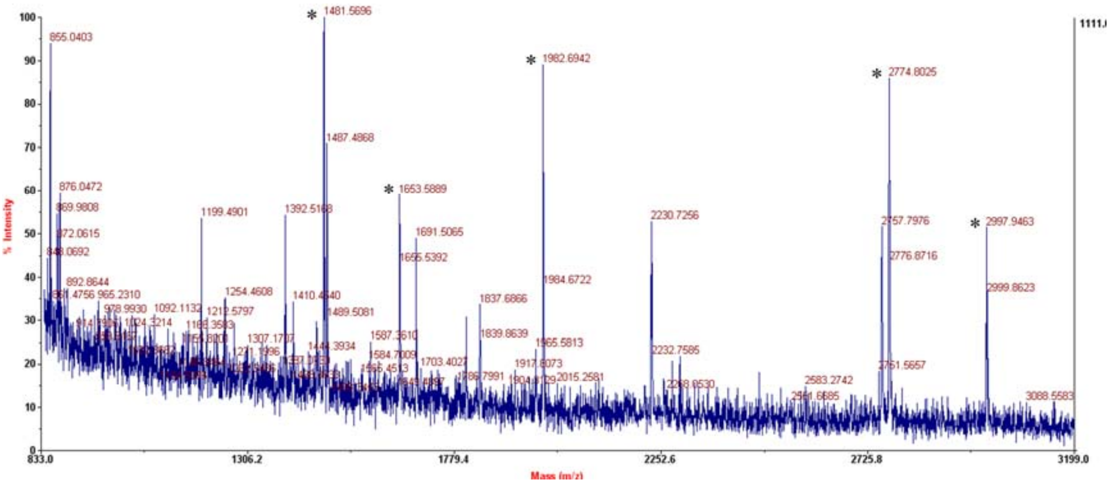

B. Peptides detected by MALDI-TOF-MS

Peptide	Amino acid sequence	$[M+H]^+$	Matched
4 - 25	GPAVGIDLGTTYSCVGVFQHGK	2262.6820	+
57 - 72	NQVAMNPTNTVFDAKR	1805.5470	+
89 - 102	HWPFMVVNDAGRPK	1653.5380	+
138 - 155	TVTNAVVTVPAYFNDSQR	1981.6220	+
273 - 299	TLSSSTQASIEIDSLYEGIDFYTSITR	2996.9690	+
300 - 311	ARFEELNADLFR	1480.4660	+
424 - 447	QTQTFTTYSDNQPGVLIQVYEGER	2773.8670	+

C. Matched peptides (Bold Red) in the HSC70 amino acid sequence

1 MSK**GPAVGID LGTTYSCVGV FQHGK**VEIIA NDQGNRTTPS YVAFTDTERL
51 IGDAAK**NQVA MNPTNTVFDA KR**LIGRRFDD AVVQSDMK**HW PFMVVNDAGR**
101 **PK**VQVEYKGE TKSFYPEEVS SMVLTKMKEI AEAYLGK**TVT NAVVTVPAYF**
151 **NDSQR**QATKD AGTIAGLNVL RIINEPTAAA IAYGLDKKVG AERNVLIFDL
201 GGGTFDVSIL TIEDGIFEVK STAGDTHLGG EDFDNRMVNH FIAEFKRKHK
251 KDISENKRAV RRLRTACERA KR**TLSSSTQA SIEIDSLYEG IDFYTSITRA**
301 **RFEELNADLF R**GTLDPVEKA LRDAKLDKSQ IHDIVLVGGS TRIPKIQKLL
351 QDFFNGKELN KSINPDEAVA YGAAVQAAIL SGDKSENVQD LLLLDVTPLS
401 LGIETAGGVM TVLIKRNTTI PTK**QTQTFTT YSDNQPGVLI QVYEGER**AMT
451 KDNNLLGKFE LTGIPPAPRG VPQIEVTFDI DANGILNVSA VDKSTGKENK
501 ITITNDKGRL SKEDIERMVQ EAEKYKAEDE KQRDKVSSKN SLESYAFNMK
551 ATVEDEKLQG KINDEDKQKI LDKCNEIISW LDKNQTAEKE EFEHQQKELE
601 KVCNPIITKL YQSAGGMPGG MPGGFPGGGA PPSGGASSGP TIEEVD

A. MALDI-TOF MS spectrum (spot 4)

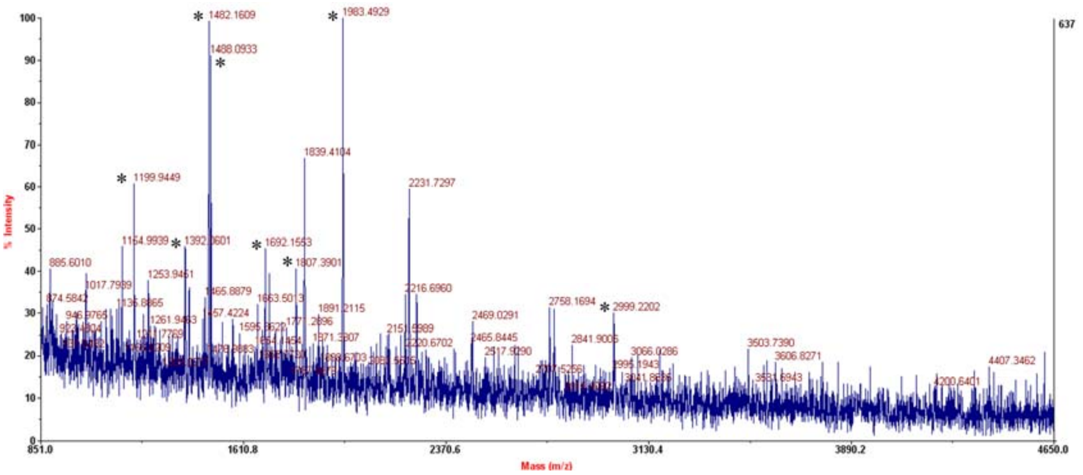

B. Peptides detected by MALDI-TOF-MS

Peptide	Amino acid sequence	$[M+H]^+$	Matched
37 - 49	TTPSYVAFTDTER	1488.0990	+
57 - 72	NQVAMNPTNTVFDAKR	1806.3720	+
138 - 155	TVTNAVVTVPAYFNDSQR	1982.5020	+
160 - 171	DAGTIAGLNVLR	1199.9640	+
221 - 236	STAGDTHLGGEDFDNR	1692.1830	+
237 - 247	MVNHFIAEFKR	1392.0750	+
273 - 299	TLSSSTQASIEIDSLYEGIDFYTSITR	2998.1880	+
300 - 311	ARFEELNADLFR	1481.1550	+
326 - 342	LDKSQIHDIVLVGGSTR	1838.4920	+
362 - 384	SINPDEAVAYGAAVQAAILSGDK	2260.7990	+
424 - 447	QTQTFTTYSDNQPGVLIQVYEGER	2775.0770	+

C. Matched peptides (Bold Red) in the HSC70 amino acid sequence

1 MSKGPAVGID LGTTYSCVGV FQHGKVEIIA NDQGNR**TTPS YVAFTDTER**L
51 IGDAAK**NQVA MNPTNTVFDA KR**LIGRRFDD AVVQSDMKHW PFMVVNDAGR
101 PKVQVEYKGE TKSFYPEEVS SMVLTKMKEI AEAYLGK**TVT NAVVTVPAYF**
151 **NDSQR**QATK**D AGTIAGLNVL R**IINEPTAAA IAYGLDKKVG AERNVLIFDL
201 GGGTFDVSIL TIEDGIFEVK **STAGDTHLGG EDFDNRMVNH FIAEFKR**KHK
251 KDISENKRAV RRLRTACERA KR**TLSSSTQA SIEIDSLYEG IDFYTSITRA**
301 **RFEELNADLF R**GTLDPVEKA LRDAK**LDKSQ IHDIVLVGGS TR**IPKIQKLL
351 QDFFNGKELN K**SINPDEAVA YGAAVQAAIL SGDK**SENVQD LLLLDVTPLS
401 LGIETAGGVM TVLIKRNTTI PTK**QTQTFTT YSDNQPGVLI QVYEGER**AMT
451 KDNNLLGKFE LTGIPPAPRG VPQIEVTFDI DANGILNVSA VDKSTGKENK
501 ITITNDKGRL SKEDIERMVQ EAEKYKAEDE KQRDKVSSKN SLESYAFNMK
551 ATVEDEKLQG KINDEDKQKI LDKCNEIISW LDKNQTAEKE EFEHQQKELE
601 KVCNPIITKL YQSAGGMPGG MPGGFPGGGA PPSGGASSGP TIEEVD

A. MALDI-TOF MS spectrum (spot 5)

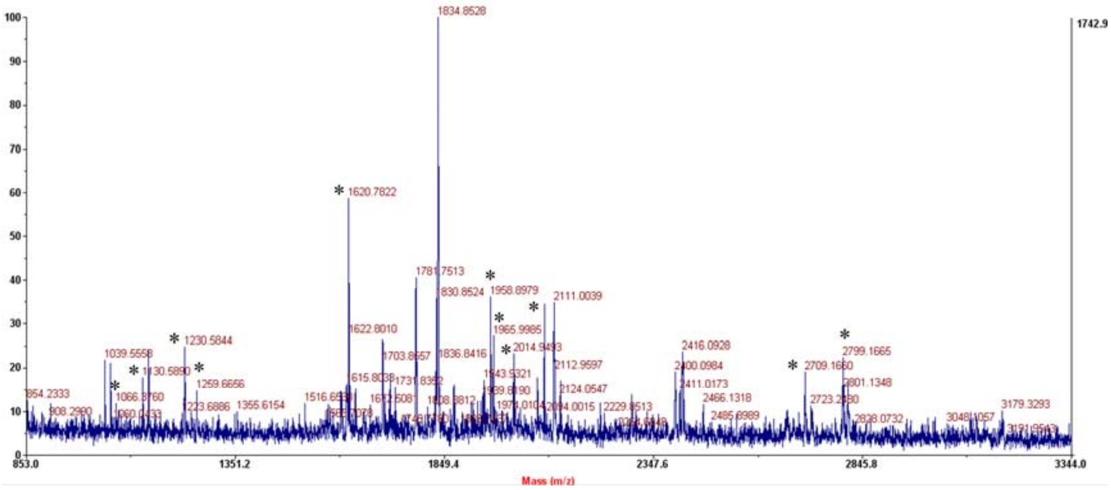

B. Peptides detected by MALDI-TOF-MS

Peptide	Amino acid sequence	$[M+H]^+$	Matched
1 - 19	MREIVHIQAGQCGNQIGAK	2109.9740	+
47 - 62	INVYYNEAAGNKYVPR	1870.8500	+
78 - 103	SGPFGQIFRPDNFVFGQSGAGNNWAK	2798.1530	+
104 - 121	GHYTEGAELVDSVLDVVR	1958.9080	+
217 - 241	LTTPTYGDLNHLVSATMSGVTTCLR	2708.1630	+
242 - 251	FPGQLNADLR	1130.5870	+
242 - 252	FPGQLNADLRK	1258.6740	+
253 - 262	LAVNMVPFPR	1143.6220	+
263 - 276	LHFFMPGFAPLTSR	1620.8000	+
263 - 276	LHFFMPGFAPLTSR	1636.7920	+
310 - 318	YLTVAAIFR	1053.5920	+
363 - 380	MSATFIGNSTAIQELFKR	2013.9550	+
381 - 390	ISEQFTAMFR	1229.6140	+

C. Matched peptides (Bold Red) in the Tubulin amino acid sequence

1 **MREIVHIQAG QCGNQIGAK**F WEVISDEHGI DPTGSYHGDS DLQLER**INVY**
51 **YNEAAGNKYV PR**AILVDLEP GTMDSVR**SGP FGQIFRPDNF VFGQSGAGNN**
101 **WAKGHYTEGA ELVDSVLDVV RK**ESESCDCL QGFQLTHSLG GGTGSGMGTL
151 LISKIREEYP DRIMNTFSVM PSPKVSDTVV EPYNATLSVH QLVENTDETY
201 SIDNEALYDI CFRTLK**LTTP TYGDLNHLVS ATMSGVTTCL RFPGQLNADL**
251 **RKLAVNMVPF PRLHFFMPGF APLTSR**GSQQ YRALTVPELT QQMFDSKNMM
301 AACDPRHGR**Y LTVAAIFR**GR MSMKEVDEQM LNVQNKNSSY FVEWIPNNVK
351 TAVCDIPPRG LK**MSATFIGN STAIQELFKR ISEQFTAMFR** RKAFLHWYTG
401 EGMDEMEFTE AESNMNDLVS EYQQYQDATA DEQGEFEEEE GEDEA

A. MALDI-TOF MS spectrum (spot 6)

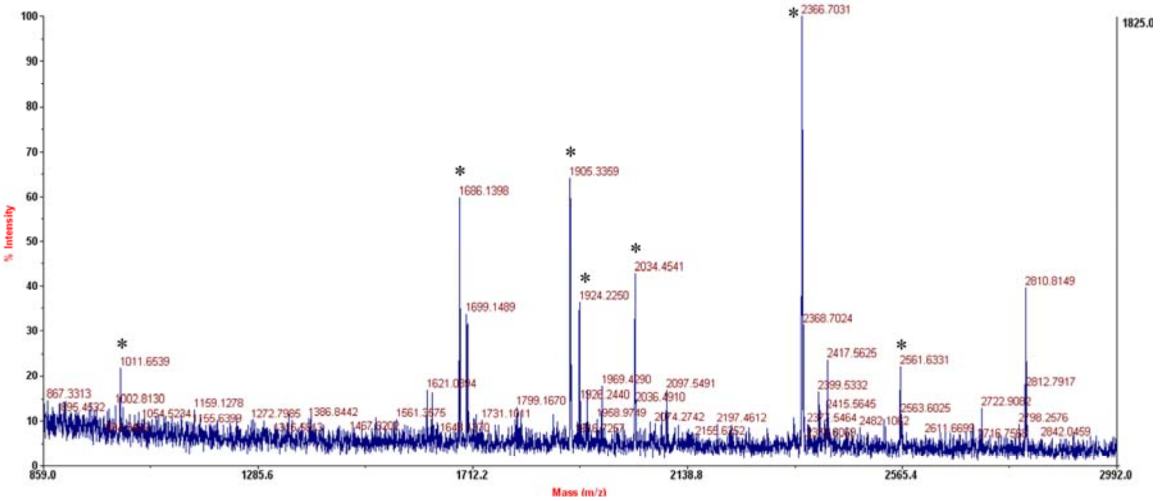

B. Peptides detected by MALDI-TOF-MS

Peptide	Amino acid sequence	$[M+H]^+$	Matched
97 - 121	LVQDVANNTNEEAGDGTTTATVLAR	2560.6330	+
134 - 142	GANPVEIRR	1011.6500	+
206 - 221	TLNDELEIIEGMKFDR	1923.2330	+
206 - 221	TLNDELEIIEGMKFDR	1939.2330	+
250 - 268	KISSVQSIVPALEIANAHR	2033.4540	+
251 - 268	ISSVQSIVPALEIANAHR	1905.3260	+
269 - 290	KPLVIIAEDVDGEALSTLVLNR	2365.7010	+
345 - 359	VGEVIVTKDDAMLLK	1631.1390	+
430 - 446	AAVEEGIVLGGGCALLR	1685.1500	+

C. Matched peptides (Bold Red) in the HSP60 amino acid sequence

1 MLRLPTVLRQ MRPVSRALAP HLTRAYAKDV KFGADARALM LQGVDLLADA
51 VAVTMGPKGR TVIIEQSWGS PKVTKDGVTV AKSIDLKDKY KNIGAK**LVQD**
101 **VANNTNEEAG DGTTTATVLA R**SIAKEGFEK ISK**GANPVEI RR**GVMLAVDA
151 VIAELKKQSK PVTTPEEIAQ VATISANGDK DIGNIISDAM KKVGRKGVIT
201 VKDGK**TLNDE LEIIEGMKFD R**GYISPYFIN TSKGQKCEFQ DAYVLLSEK**K**
251 **ISSVQSIVPA LEIANAHRKP LVIIAEDVDG EALSTLVLNR** LKVGLQVVAV
301 KAPGFGDNRK NQLKDMAIAT GGAVFGEEGL NLNLEDVQAH DLGK**VGEVIV**
351 **TKDDAMLLK**G KGDKAHIEKR IQEITEQLDI TTSEYEKEKL NERLAKLSDG
401 VAVLKVGGTS DVEVNEKKDR VTDALNATR**A AVEEGIVLGG GCALLR**CIPA
451 LDSLKPANED QKIGIEIIKR ALKIPAMTIA KNAGVEGSLI VEKILQSSSE
501 VGYDAMLGDF VNMVEKGIID PTKVVRTALL DAAGVASLLT TAEAVVTEIP
551 KEEKDPGMGA MGGMGGGMGG GMF

A. MALDI-TOF MS spectrum (spot 7)

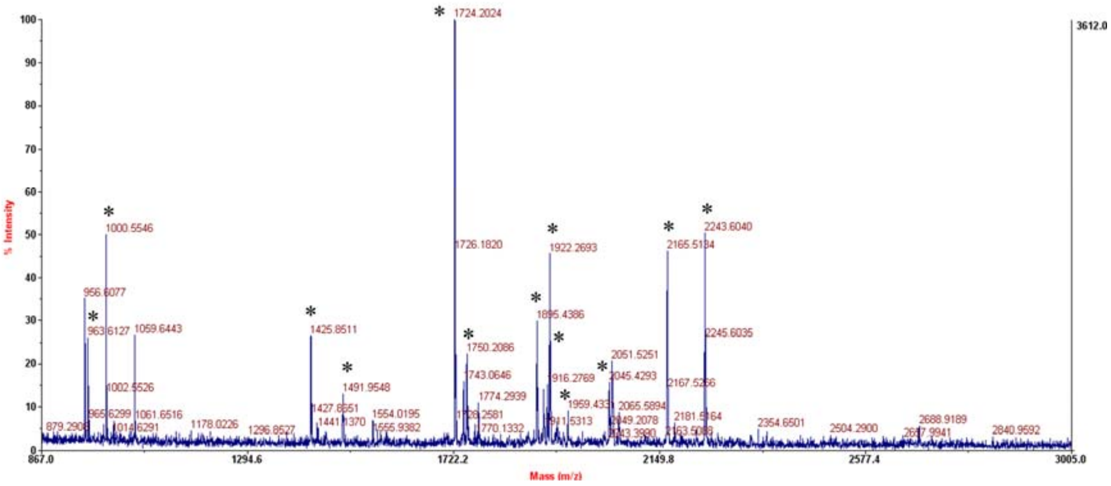

B. Peptides detected by MALDI-TOF-MS

Peptide	Amino acid sequence	$[M+H]^+$	Matched
69-76	MVEGFFDR	1000.5540	+
108 - 123	IIKPCNHVLSLSFPIR	1894.4380	+
125 - 136	DDGSWEVIEGYR	1425.8600	+
137 - 143	AQHSQHR	863.4720	+
212 - 231	KGFIGPGIDVPAPDMSTGER	2044.4190	+
213 - 231	GFIGPGIDVPAPDMSTGER	1916.2980	+
303 - 318	TFVVQGFGNVGLHSMR	1749.2120	+
347 - 363	ELEDFKLQHGSILGFPK	1958.3770	+
400 - 420	IIAEGANGPTTPEADKIFLER	2242.5740	+
445 - 453	NLNHVSYGR	1059.6440	+
454 - 460	LTFKYER	956.6030	+
461 - 476	DSNYHLLMSVQESLER	1921.2680	+
481 - 496	HGGTIPVVPTAEFQDR	1724.1860	+
497 - 516	ISGASEKDIVHSGLAYTMER	2164.4730	+
504 - 516	DIVHSGLAYTMER	1491.9690	+
528 - 535	YNLGLDLR	963.6170	+

C. Matched peptides (Bold) in the GDH amino acid sequence

**1**	MYRRLGEVLL	LSRAGPAALG	SAAADSAALL	GWARGQPSAV	PQPGLTPVAR
**51**	RHYSEAATDR	EDDPNFFK**MV**	**EGFFDR**GASI	VEDKLVEDLK	TRENEEQKRN
**101**	RVRGILR**IIK**	**PCNHVLSLSF**	**PIRRDDGSWE**	**VIEGYRAQHS**	**QHR**TPCKGGI
**151**	RYSTDVSVDE	VKALASLMTY	KCAVVDVPFG	GAKAGVKINP	KNYTDNELEK
**201**	ITRRFTMELA	K**KGFIGPGID**	**VPAPDMSTGE**	**REMSWIADTY**	**ASTIGHYDIN**
**251**	**AHACVTGKPI**	**SQGGIHGR**IS	ATGRGVFHGI	ENFINEASYM	SILGMTPGLG
**301**	DK**TFVVQGFG**	**NVGLHSMR**YL	HRFGAKCVGV	GESDGSIWNP	DGIDPK**ELED**
**351**	**FKLQHGSILG**	**FPK**AKVYEGS	**ILEADCDILI**	**PAASEK**QLTK	SNAPRVKAK**I**

401 IAEGANGPTT PEADKIFLER NIMVIPDLYL NAGGVTVSYF EWLK**NLNHVS**
451 YGRLTFKYER DSNYHLLMSV QESLERKFGK **HGGTIPVVPT AEFQDRISGA**
501 SEKDIVHSGL AYTMERSARQ IMRTAMK**YNL GLDLRTAAYV NAIEKVFK**VY
551 NEAGVTFT

A. MALDI-TOF MS spectrum (spot 8)

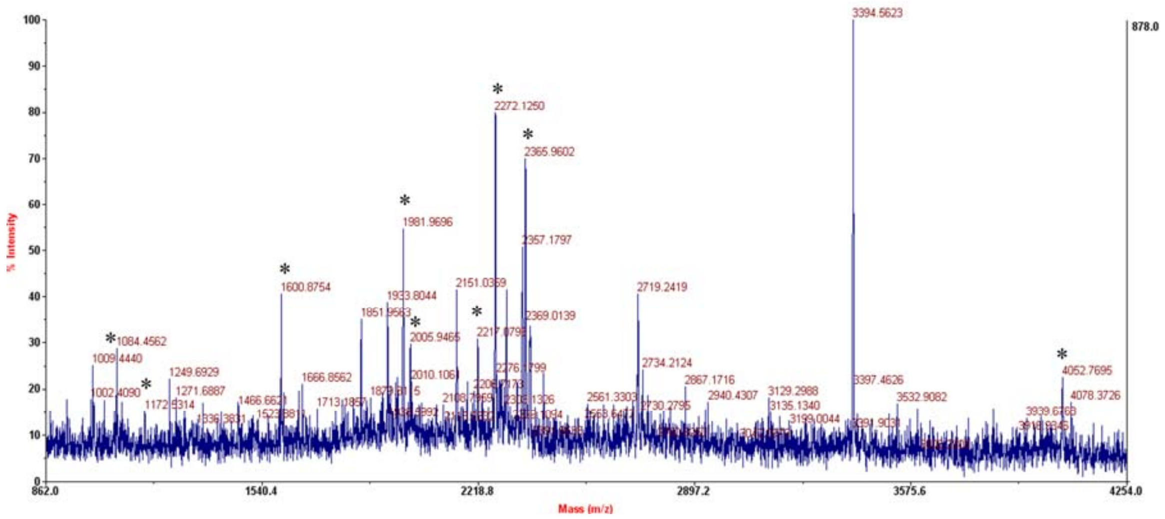

B. Peptides detected by MALDI-TOF-MS

Peptide	Amino acid sequence	[M+H]+	Matched
83 - 97	ASLQNLLSASQAQLR	1599.8770	+
106-142	AFTSQASATMHLPATIGDYTDFYSSLQHATNVGIMFR	4049.7500	+
143-162	GKENALLPNWLHLPVGYHGR	2271.2040	+
163-174	ASSVVVSGTPIR	1172.5610	+
175-194	RPMGQMRPDNSKPPVYGASK	2216.0580	+
195-211	RLDMELEMAFFVGPGNR	1981.9410	+
221-237	AQEHIFGMVLMNDWSAR	2004.9210	+
372-381	AIDVGQGQTR	1044.4740	+
382-402	TFLLDGDEVIITGHCQGDGYR	2366.0740	+

C. Matched peptides (Bold Red) in the Fumarylacetoacetase amino acid sequence

1 MSFIPVAEDS DFPIQNLPYG VFSTQSNPKP RIGVAIGDQI LDLSVIKHLF
51 TGPVLSKHQH VFDETTLNSF MGLGQAAWKE AR**ASLQNLLS ASQAQLR**DDK
101 ELRQR**AFTSQ ASATMHLPAT IGDYTDFYSS LQHATNVGIM FRGKENALLP**
151 **NWLHLPVGYH GRASSVVVSG TPIRRPMGQM RPDNSKPPVY GASKRLDMEL**
201 **EMAFFVGPGN R**FGEPIPISK **AQEHIFGMVL MNDWSAR**DIQ QWEYVPLGPF
251 LGKSFGTTIS PWVVPMDALM PFVVPNPKQD PKPLPYLCHS QPYTFDINLS
301 VALKGEGMSQ AATICRSNFK HMYWTILQQL THHSVNGCNL RPGDLLASGT
351 ISGSDPESFG SMLELSWKGT K**AIDVGQGQT RTFLLDGDEV IITGHCQGDG**
401 **YR**VGFGQCAG KVLPALSPA

A. MALDI-TOF MS spectrum (spot 9)

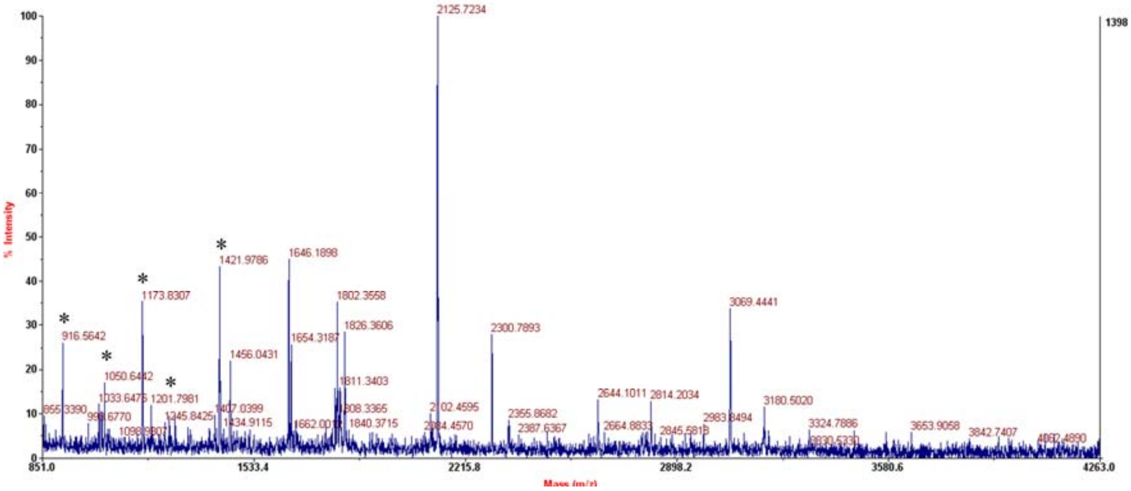

B. Peptides detected by MALDI-TOF-MS

Peptide	Amino acid sequence	$[M+H]^+$	Matched
15-22	ELSDIALR	916.5810	+
61-69	QVLFSADDR	1050.6480	+
61-71	QVLFSADDRVK	1277.9280	+
102-111	GILVGIKVDK	1041.7720	+
305-315	ALQASALSAWR	1173.8340	+
319-331	DNAGAATEEFIKR	1421.9770	+
331-342	RAEMNGLAAQGK	1245.8630	+

C. Matched peptides (Bold Red) in the Fructose-bisphosphate aldolase C amino acid sequence

1 MPHSYPALSA EQKK**ELSDIA LR**IVAPGKGI LAADESVGSM AKRLSQIGVE
51 NTEENRRLYR **QVLFSADDRV K**KCIGGVIFF HETLYQKDDN GVPFVRTIQE
101 K**GILVGIKVD K**GVVPLAGTD GETTTQGLDG LLERCAQYKK DGADFAKWRC
151 VLKISDRTPS ALAILENANV LARYASICQQ NGIVPIVEPE ILPDGDHDLK
201 RCQFVTEKVL AAVYKALSDH HVYLEGTLLK PNMVTPGHAC PIKYSPEEIA
251 MATVTALRRT VPPAVPGVTF LSGGQSEEEA SLNLNAINRC SLPRPWALTF
301 SYGR**ALQASA LSAWR**GQR**DN AGAATEEFIK RAEMNGLAAQ GK**YEGSGDGG
351 AAAQSLYVAN HAY

A. MALDI-TOF MS spectrum (spot10)

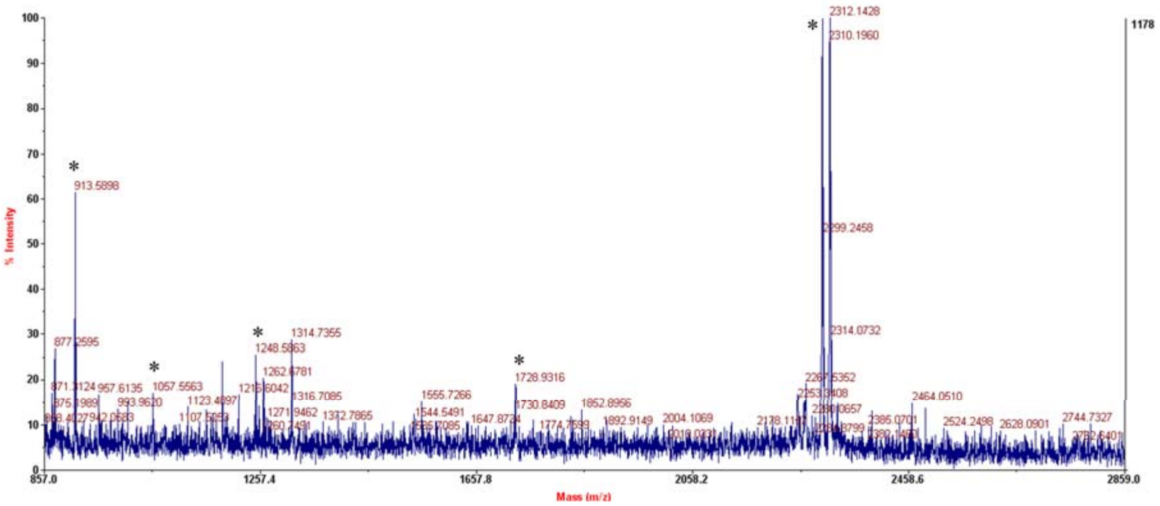

B. Peptides detected by MALDI-TOF-MS

Peptide	Amino acid sequence	$[M+H]^+$	Matched
92-100	IVVVTAGVR	913.5910	+
92-107	IVVVTAGVRQQEGESR	1727.8920	+
159-170	VIGSGCNLDSAR	1248.5780	+
171-178	FRYLMAEK	1057.5650	+
280-299	GMYGIENEVFLSLPCILNAR	2296.1350	+
309-318	LKDDEVAQLR	1186.6320	+

C. Matched peptides (Bold Red) in the LDH amino acid sequence

1 MATLKEKLIA PVADDETAVP NNKITVVGVG QVGMACAISI LGKSLADELA
51 LVDVLEDKLK GEMMDLQHGS LFLQTPKIVA DKDYSVTANS K**IVVVTAGVR**
101 **QQEGESR**LNL VQRNVNVFKF IIPQIVKYSP DCTIIVVSNP VDILTYVTWK
151 LSGLPKHR**VI GSGCNLDSAR FRYLMAEK**LG IHPSSCHGWI LGEHGDSSVA
201 VWSGVNVAGV SLQELNPEMG TDNDSENWKE VHKMVVDSAY EVIKLKGYTN
251 WAIGLSVADL IESMLKNLSR IHPVSTMVK**G MYGIENEVFL SLPCILNAR**G
301 LTSVINQK**LK DDEVAQLR**KS ADTLWDIQKD LKDL

A. MALDI-TOF MS spectrum (spot 11)

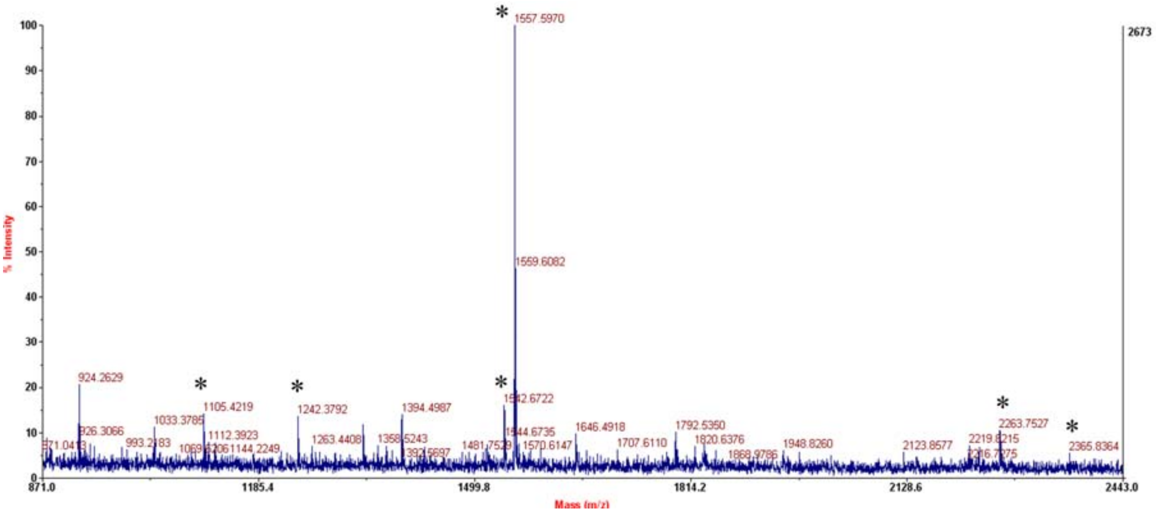

B. Peptides detected by MALDI-TOF-MS

Peptide	Amino acid sequence	$[M+H]^+$	Matched
70-78	RQDLFIVSK	1105.4340	+
156-169	AIGVSNFNPLQIER	1557.5990	+
244-256	TTAQVLIRFPIQR	1542.6660	+
276-294	FRYLMAEK	2263.7400	+
280-299	VFDFELSNEDMATLLSYNR	2296.1350	+
307-316	HKDYPFHAEV	1242.3780	+

C. Matched peptides (Bold Red) in the AR amino acid sequence

1 MASHLELNNG TKMPTLGLGT WKSPPGQVTE AVKVAIDMGY RHIDCAQVYQ
51 NEKEVGVALQ EKLKEQVVK**R QDLFIVSK**LW CTFHDQSMVK GACQKTLSDL
101 QLDYLDLYLI HWPTGFKPGP DYFPLDASGN VIPSDTDFVD TWTAMEQLVD
151 EGLVK**AIGVS NFNPLQIER**I LNKPGLKYKP AVNQIECHPY LTQEKLIEYC
201 HCKGIVVTAY SPLGSPDRPW AKPEDPSLLE DPRIKEIAAK YNK**TTAQVLI**
251 **RFPIQR**NLVV IPKSVTPARI AENFK**VFDFE LSNEDMATLL SYNR**NWRVCA
301 LMSCAK**HKDY PFHAEV**

A. MALDI-TOF MS spectrum (spot 12)

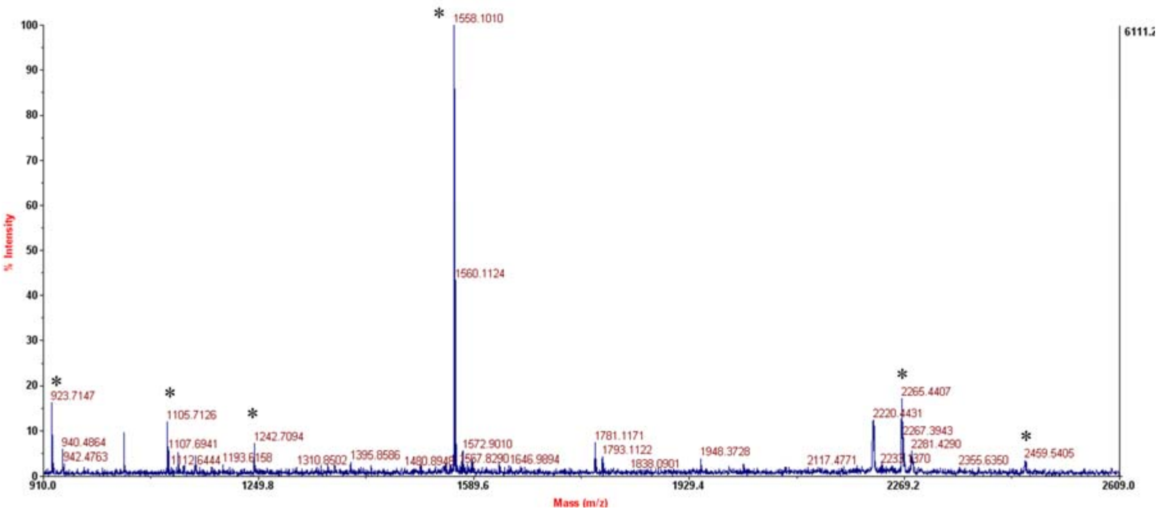

B. Peptides detected by MALDI-TOF-MS

Peptide	Amino acid sequence	$[M+H]^+$	Matched
34-41	VAIDMGYR	924.4990	+
42-62	HIDCAQVYQNEKEVGVALQEK	2458.5160	+
70-78	RQDLFIVSK	1105.7080	+
156-169	AIGVSNFNPLQIER	1558.0120	+
196-203	LIEYCHCK	1122.5820	+
244-251	TTAQVLIR	901.5900	+
276-294	VFDFELSNEDMATLLSYNR	2264.3420	+
307-316	HKDYPFHAEV	1242.7110	+

C. Matched peptides (Bold Red) in the AR amino acid sequence

1 MASHLELNNG TKMPTLGLGT WKSPPGQVTE AVK**VAIDMGY RHIDCAQVYQ**
51 **NEKEVGVALQ EK**LKEQVVK**R QDLFIVSK**LW CTFHDQSMVK GACQKTLSDL
101 QLDYLDLYLI HWPTGFKPGP DYFPLDASGN VIPSDTDFVD TWTAMEQLVD
151 EGLVK**AIGVS NFNPLQIER**I LNKPGLKYKP AVNQIECHPY LTQEK**LIEYC**
201 **HCK**GIVVTAY SPLGSPDRPW AKPEDPSLLE DPRIKEIAAK YNK**TTAQVLI**
251 **R**FPIQRNLVV IPKSVTPARI AENFK**VFDFE LSNEDMATLL SYNR**NWRVCA
301 LMSCAK**HKDY PFHAEV**

A. MALDI-TOF MS spectrum (spot 13)

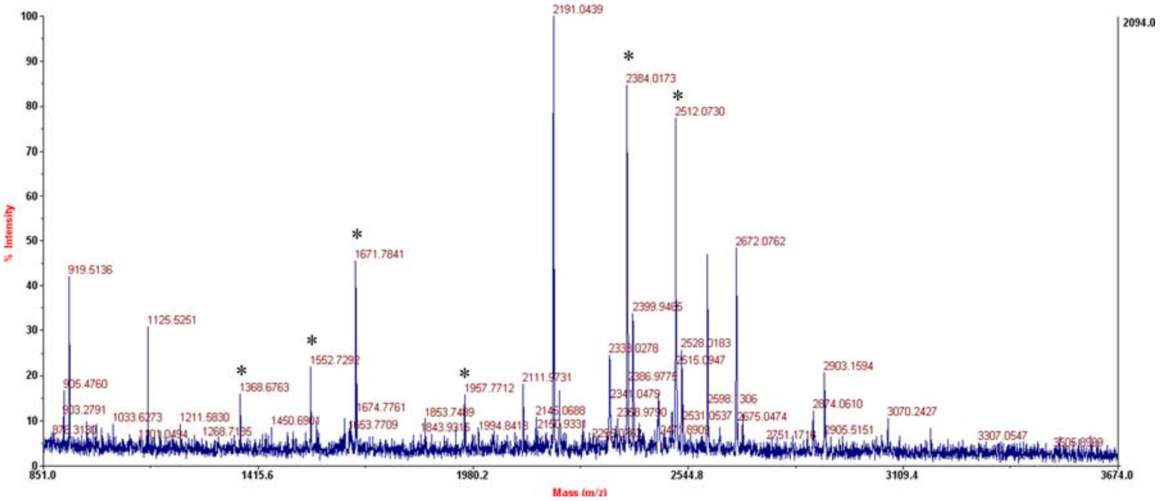

B. Peptides detected by MALDI-TOF-MS

Peptide	Amino acid sequence	$[M+H]^+$	Matched
9-16	RVLVYGGR	924.4990	+
53 -70	MTDSFTEQADQVTAEVGK	1956.7540	+
136-151	AALDGTPGMIGYGMAK	1552.7290	+
152-164	GAVHQLCQSLAGK	1368.6720	+
165-188	NSGMPSGAAAIAVLPVTLDTPMNR	2382.9970	+
165-189	NSGMPSGAAAIAVLPVTLDTPMNRK	2511.0690	+
218-233	RPNSGSLIQVVTTDGK	1671.8010	+

C. Matched peptides (Bold Red) in the DHPR amino acid sequence

1 MAASGEAR**RV LVYGGR**GALG SRCVQAFRAR NWWVASIDVV ENEEASASVI

51 VK**MTDSFTEQ ADQVTAEVGK** LLGDQKVDAI LCVAGGWAGG NAKSKSLFKN

101 CDLMWKQSIW TSTISSHLAT KHLKEGGLLT LAGAK**AALDG TPGMIGYGMA**

151 **KGAVHQLCQS LAGKNSGMPS GAAAIAVLPV TLDTPMNRK**S MPEADFSSWT

201 PLEFLVETFH DWITGNK**RPN SGSLIQVVTT DGK**TELTPAY F

A. MALDI-TOF MS spectrum (spot 14)

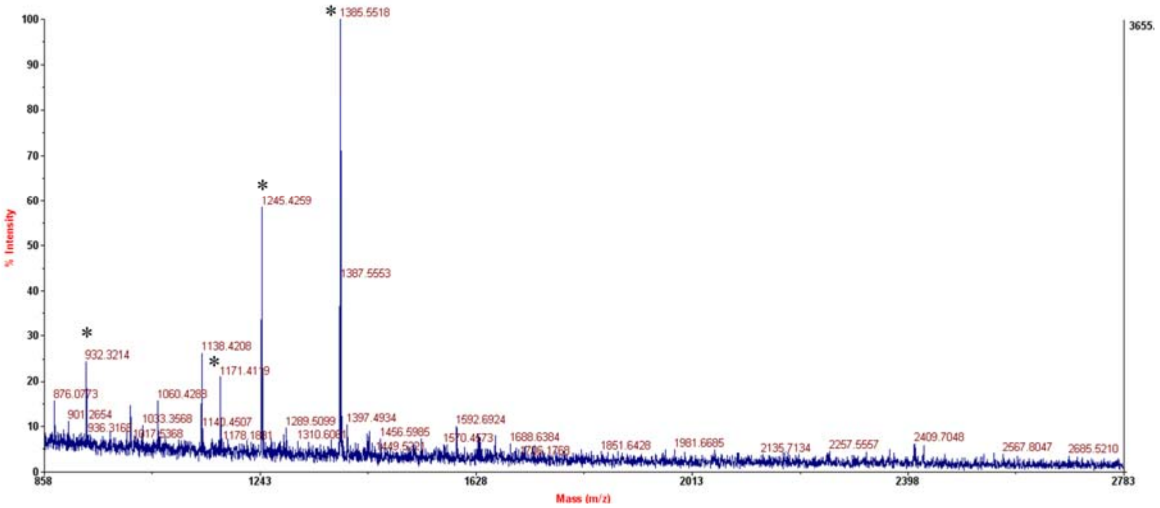

B. Peptides detected by MALDI-TOF-MS

Peptide	Amino acid sequence	$[M+H]^+$	Matched
2-13	PGKPVLHYFDGR	1385.6230	+
130-138	NRYFPAFEK	1171.5620	+
142-152	SHGQDYLVGNR	1245.5900	+
142-155	SHGQDYLVGNRLSR	1601.6710	+
197-204	FLQPGSQR	932.4410	+

C. Matched peptides (Bold Red) in the GSTA3 amino acid sequence

1 M**PGKPVLHYF DGR**GRMEPIR WLLAAAGVEF EEQFLKTRDD LARLRNDGSL
51 MFQQVPMVEI DGMKLVQTRA ILNYIATKYN LYGKDMKERA LIDMYAEGVA
101 DLDEIVLHYP YIPPGEKEAS LAKIKDKAR**N RYFPAFEK**VL K**SHGQDYLVG**
151 **NRLSR**ADVYL VQVLYHVEEL DPSALANFPL LKALRTRVSN LPTVKK**FLQP**
201 **GSQR**KPLEDE KCVESAVKIF S

A. MALDI-TOF MS spectrum (spot 15)

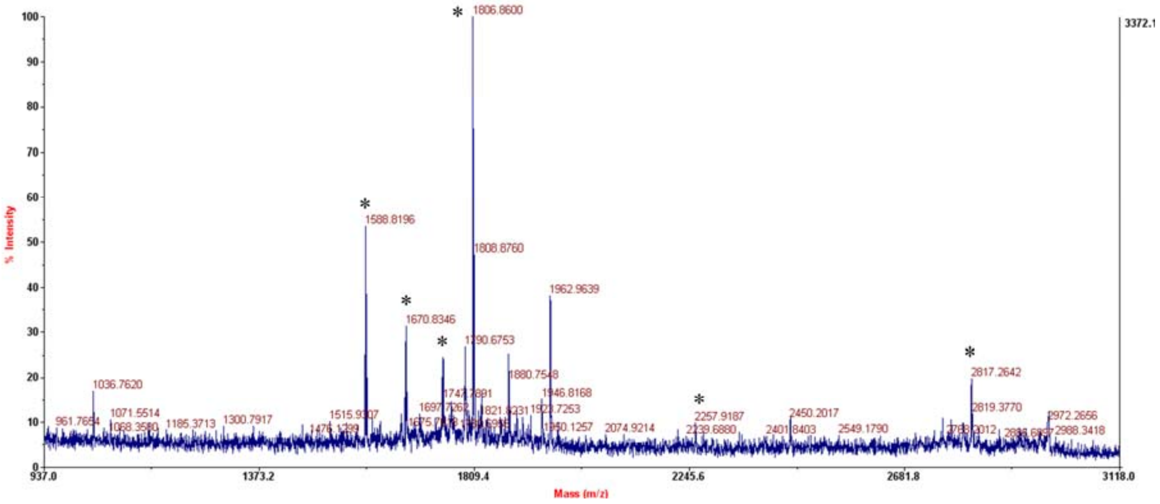

B. Peptides detected by MALDI-TOF-MS

Peptide	Amino acid sequence	$[M+H]^+$	Matched
32-45	MLQLVEESKDAGIR	1588.8210	+
46-59	TLVMLDEQGEQLER	1660.8090	+
104-119	AWGNNQDGVVASQPAR	1669.7700	+
120-135	VVDEREQMAISGGFIR	1806.8720	+
137-161	VTNDARENEMDENLEQVSGIIGNLR	2816.2520	+
162-176	HMALDMGNEIDTQNR	1744.7850	
162-180	HMALDMGNEIDTQNRQIDR	2256.9290	

C. Matched peptides (Bold Red) in the SNP25 amino acid sequence

1 MAEDADMRNE LEEMQRRADQ LADESLESTR R**MLQLVEESK DAGIRTLVML**
51 **DEQGEQLER**I EEGMDQINKD MKEAEKNLTD LGKFCGLCVC PCNKLKSSDA
101 YKK**AWGNNQD GVVASQPARV VDEREQMAIS GGFIR**R**VTND ARENEMDENL**
151 **EQVSGIIGNL RHMALDMGNE IDTQNRQIDR** IMEKADSNKT RIDEANQRAT
201 KMLGSG

A. MALDI-TOF MS spectrum (spot 16)

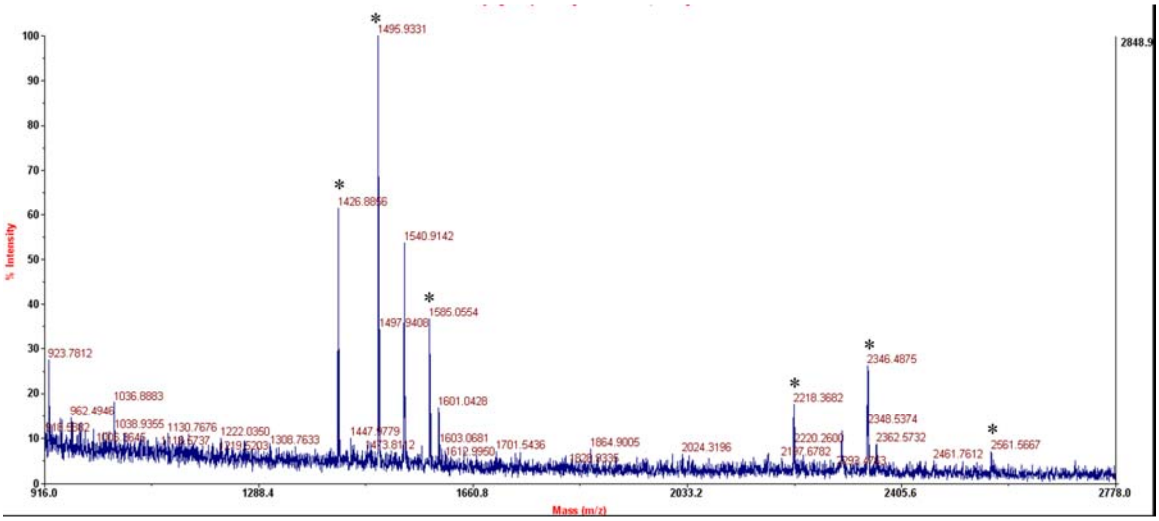

B. Peptides detected by MALDI-TOF-MS

Peptide	Amino acid sequence	$[M+H]^+$	Matched
32-44	YGYTHLSTGDLLR	1495.9390	+
64 -77	GELVPLETVLDMLR	1585.0450	+
108-128	KIAQPTLLLYVDAGPETMTQR	2345.4940	+
109-128	IAQPTLLLYVDAGPETMTQR	2217.3710	+
156-167	ATEPVISFYDKR	1425.8930	+
172-194	KVNAEGSVDTVFSQVCTYLDSLK	2560.5700	+

C. Matched peptides (Bold) in the AK amino acid sequence

1 MEDKLKKAKI IFVVGGPGSG KGTQCEKIVQ K**YGYTHLSTG DLLR**AEVSSG
51 SSRGKMLSSI MEK**GELVPLE TVLDMLR**DAM LAKVDSSNGF LIDGYPREVK
101 QGEEFER**KIA QPTLLLYVDA GPETMTQR**LL KRGETSGRVD DNEETIKKRL
151 ETYYK**ATEPV ISFYDKR**GIV R**KVNAEGSVD TVFSQVCTYL DSLK**

A. MALDI-TOF MS spectrum (spot 17)

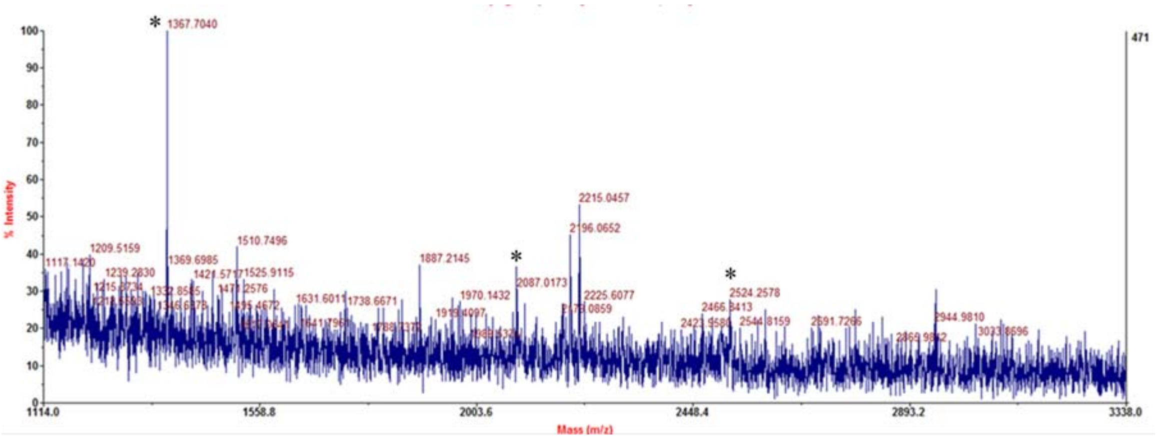

B. Peptides detected by MALDI-TOF-MS

Peptide	Amino acid sequence	$[M+H]^+$	Matched
72-92	HGGPADEERHVGDLGNVAAGK	2085.9560	+
93-116	DGVANVSIEDRVISLSGEHSIIGR	2523.1270	+
104-116	VISLSGEHSIIGR	1367.7090	+

C. Matched peptides (Bold) in the SOD amino acid sequence

1 MAMKAVCVLK GDGPVQGVIH FEQKASGEPV VVSGQITGLT EGEHGFHVHQ
51 YGDNTQGCTT AGPHFNPHSK K**HGGPADEER HVGDLGNVAA GKDGVANVSI**
101 **EDRVISLSGE HSIIGR**TMVV HEKQDDLGKG GNEESTKTGN AGSRLACGVI
151 GIAQ

A. MALDI-TOF MS spectrum (spot 18)

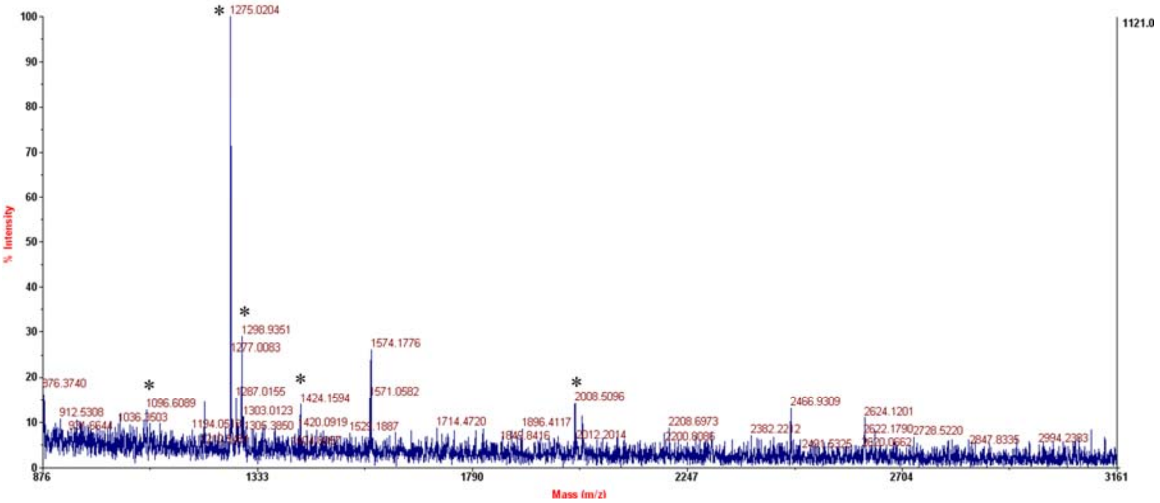

B. Peptides detected by MALDI-TOF-MS

Peptide	Amino acid sequence	$[M+H]^+$	Matched
19-31	VNPDDVGGEALGR	1298.9430	+
32-41	LLVVYPWTQR	1275.0190	+
42-60	YFDSFGDLSSASAIMGNPK	2007.5030	+
68 -77	VINAFNDGLK	1090.8120	
134-147	VVAGVASALAHKYH	1423.1460	

C. Matched peptides (Bold Red) in the Hemoglobin subunit beta-1 amino acid sequence

1 MVHLTDAEKA AVNGLWGK**VN PDDVGGEALG RLLVVYPWTQ RYFDSFGDLS**
51 **SASAIMGNPK** VKAHGKK**VIN AFNDGLKHLD NLK**GTFAHLS ELHCDKLHVD
101 PENFRLLGNM IVIVLGHHLG KEFTPCAQAA FQK**VVAGVAS ALAHKYH**
